# The microbiota–gut–brain axis in Huntington's disease: pathogenic mechanisms and therapeutic targets

**DOI:** 10.1111/febs.17102

**Published:** 2024-03-01

**Authors:** Millicent N. Ekwudo, Carolina Gubert, Anthony J. Hannan

**Affiliations:** ^1^ Florey Institute of Neuroscience and Mental Health University of Melbourne Parkville Australia; ^2^ Department of Anatomy and Physiology University of Melbourne Parkville Australia

**Keywords:** diet, gut dysbiosis, gut‐brain axis, Huntington's disease, microbiome, microbiota, mycobiome, neurodegeneration

## Abstract

Huntington's disease (HD) is a currently incurable neurogenerative disorder and is typically characterized by progressive movement disorder (including chorea), cognitive deficits (culminating in dementia), psychiatric abnormalities (the most common of which is depression), and peripheral symptoms (including gastrointestinal dysfunction). There are currently no approved disease‐modifying therapies available for HD, with death usually occurring approximately 10–25 years after onset, but some therapies hold promising potential. HD subjects are often burdened by chronic diarrhea, constipation, esophageal and gastric inflammation, and a susceptibility to diabetes. Our understanding of the microbiota–gut–brain axis in HD is in its infancy and growing evidence from preclinical and clinical studies suggests a role of gut microbial population imbalance (gut dysbiosis) in HD pathophysiology. The gut and the brain can communicate through the enteric nervous system, immune system, vagus nerve, and microbiota‐derived‐metabolites including short‐chain fatty acids, bile acids, and branched‐chain amino acids. This review summarizes supporting evidence demonstrating the alterations in bacterial and fungal composition that may be associated with HD. We focus on mechanisms through which gut dysbiosis may compromise brain and gut health, thus triggering neuroinflammatory responses, and further highlight outcomes of attempts to modulate the gut microbiota as promising therapeutic strategies for HD. Ultimately, we discuss the dearth of data and the need for more longitudinal and translational studies in this nascent field. We suggest future directions to improve our understanding of the association between gut microbes and the pathogenesis of HD, and other ‘brain and body disorders’.

## Graphical abstract

Disturbances of the microbiota–gut–brain axis are evident in people with Huntington's disease (HD) and in HD animal models. Here, we provide an overview of how the trillions of microorganisms residing in the gut, their secretory products, and the microbiota–gut–brain axis may contribute to HD pathogenesis. We discuss in depth the currently available therapeutic strategies targeting gut microbes, and their promising potential as non‐invasive, safe, and efficacious treatments for HD.

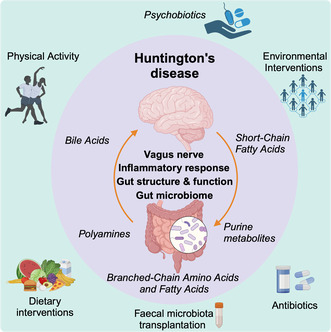

**Abbreviations**
3‐NP: 3‐nitro propionic acid
AAA: aromatic amino acid
ABX: antibiotics
AD: Alzheimer's disease
ALS: amyotrophic lateral sclerosis
ASD: autism spectrum disorder
BA: bile acid
BBB: blood–brain barrier
BCAA: branched‐chain amino acid
BCFA: branched‐chain fatty acid
BMI: body mass index
CA: cholic acid
CA: corpora amylacea
CAG: cytosine‐adenine‐guanine
CAIP: cholinergic anti‐inflammatory pathway
CART: cocaine‐ and amphetamine‐regulated transcript
CCK: cholecystokinin
CDCA: chenodeoxycholic acid
CNS: central nervous system
CRP: C‐reactive protein
CSF: cerebrospinal fluid
CXCL8: C‐X‐C motif chemokine ligand 8
EE: environmental enrichment
EEC: enteroendocrine cells
ENS: enteric nervous system
ER: endoplasmic reticulum
ET: essential tremor
EX: exercise
F/B: Firmicutes‐to‐Bacteroides
FMT: fecal microbiota transplantation
FXR: farnesoid X receptor
GABA: γ‐aminobutyric acid
GF: germ‐free
GIT: gastrointestinal tract
GLP‐1: glucagon‐like peptide 1
HD: Huntington's disease
HDGEC: Huntington's disease gene expansion carrier
HTT: huntingtin
IBD: inflammatory bowel disease
IBS: irritable bowel syndrome
IEC: intestinal epithelial cell
IF: intermittent fasting
IFN‐γ: interferon‐gamma
IL: interleukin
IL‐7R: interleukin‐7 receptor
iNOS: inducible nitric oxide synthetase
KD: ketogenic diet
KO: KEGG orthology
LPS: lipopolysaccharide
MDD: major depressive disorder
MeDi: Mediterranean diet
mHTT: mutant huntingtin
MMP: matrix metalloproteinase
MMSE: Mini‐Mental State Examination
mRNA: messenger RNA
MS: multiple sclerosis
MTM: mithramycin
ND: neurodegenerative disorder
NOD1: nucleotide‐binding oligomerization domain‐containing protein 1
PA: polyamine
PD: Parkinson's disease
PERMANOVA: permutational multivariate analysis of variance
PGP: protein gene product
PRR: pattern recognition receptors
QA: quinolinic acid
ROS: reactive oxygen species
SCFA: short‐chain fatty acid
SH: standard housing
SPF: specific pathogen‐free
STAT‐1: signal transducer and activator of transcription‐1
TFC: total functional capacity
TGF‐β1: transforming growth factor‐beta1
TJ: tight junctions
TLR: toll‐like receptor
TNF‐α: tumor necrosis factor‐alpha
TRKD: time‐restricted ketogenic diet
TUDCA: tauroursodeoxycholic acid
UDCA: ursodeoxycholic acid
VaCHT: vesicular acetylcholine transporter
VIP: vasoactive intestinal polypeptide
VNS: vagal nerve stimulation
WT: wild‐type
YAC: yeast artificial chromosome
ZO: zonula occludens

## Introduction

Huntington's disease (HD) is a devastating neurodegenerative disorder (ND) in which subjects are heavily plagued with cognitive, psychiatric, motor, gastrointestinal, and metabolic impairments [1]. Genetically, HD is caused by an autosomal inherited cytosine‐adenine‐guanine (CAG) trinucleotide repeat length expansion in the human *huntingtin* (*HTT*) gene located on the short arm of chromosome 4, at position 16.3 and encodes for a protein of 3144 amino acids, with a molecular weight of 350 kDa [2, 3]. The genetic defect results in the production of a mutant huntingtin (mHTT) protein consisting of long polyglutamine repeats. A positive correlation between repeat length and disease severity exists. A repeat range length of CAG between 10 and 35 is present in healthy populations whilst 36–39 repeats evoke low penetrance; subjects with >39 repeats will likely develop HD [4, 5]. The global prevalence of HD is approximately 5 per 100 000 [5], although founder effects can lead to much higher prevalence in particular regions and countries. Juvenile‐onset and adult‐onset HD subjects receive first diagnoses below and above 20 years of age respectively [6], with approximately 95% of cases exhibiting adult onset [7].

The polyglutamine‐expanded mutant protein (mHTT) engages in abnormal interaction with other molecules, as well as dysfunctional protein aggregation, leading to a range of molecular and cellular consequences, including endoplasmic reticulum (ER) stress, mitochondrial dysfunction, selective cell dysfunction and neuronal cell death [8, 9, 10]. Thus, HD bears similarities with other proteinopathies such as Alzheimer's disease (including Amyloid‐β and Tau proteotoxicity) and Parkinson's disease (including α‐synuclein proteotoxicity) [11]. HTT is ubiquitously expressed in the body, including the gastrointestinal tract (GIT) which begins from the esophagus and ends in the anus [12]. HD subjects are burdened with GI disruptions including diarrhea, gastroesophagitis, poor nutrient absorption, dysphagia (difficulty swallowing), weight loss, anal incontinence, and constipation [13, 14, 15].

Additionally, HD patients have significantly lower body mass index (BMI) compared to healthy controls, despite normal or increased (hyperphagic) food intake [16, 17, 18]. Other non‐motor symptoms include apathy, poor memory, hallucinations (psychosis), depression, and attentional deficits [19]. These non‐motor symptoms show overlap with Parkinson's disease (PD); however, these symptoms are more prevalent in HD and correlate with disease progression in HD [6, 20]. Importantly, increased incidence of diabetes, cardiovascular events, circadian disruptions, and sexual dysfunction are also comorbidities often seen in HD subjects [21, 22, 23].

The GIT is colonized by trillions of microbes (microbiota), including bacterial, archaeal, viral, and fungal species. The beneficial role of these gut residents in host health has been extensively demonstrated, as they promote various biological processes, including epithelial integrity and immune homeostasis, digestion of ingested food and protection against pathogenic invasion [24].

After the gut, the oral cavity is the second largest colonized portion of the human body [25]. It is vastly populated by over 770 diverse microorganisms and plays a vital role in systemic health [26]. Importantly, the oral microbiota is in close contact with the external environment, serving as a primary gateway and can influence the overall health of an individual [27]. Oral cavity‐specific microbes and their metabolites may escape to distant organ sites such as the small intestine, heart, lung, brain, and placenta and elicit inflammatory consequences [28]. However, the mechanisms through which this occurs require more delineation.

When microbiota composition shifts to a pathological state (dysbiosis), it may contribute to dysfunction and disease. The role of oral and gut microbiota dysbiosis in neurodegenerative and neuropsychiatric disorders has been explored in numerous studies using human and experimental models and has been extensively reviewed elsewhere [29, 30]. However, the potential role of oral dysbiosis in HD progression remains unexplored. Of particular relevance to the present article, several independent studies in humans and preclinical models have linked the gut microbiota to HD pathogenesis [31].

Gut dysbiosis is an alteration of the intestinal microbial profile and has been linked to the development and progression of numerous disease conditions [32]. Contributing factors to dysbiosis include leaky gut, unhealthy diet, other lifestyle factors, age, as well as medications (and other pharmacological agents consumed recreationally) [33, 34]. Importantly, gut dysbiosis has been shown to influence disease onset and progression in HD [1].

The gut and the central nervous system share a bidirectional connection [35, 36, 37]. Gene–environment‐gut microbiota interactions in HD have been demonstrated, making the gut‐brain axis in HD a new research focus, and inspiring novel microbiota‐targeted therapeutic approaches [34, 38]. This review provides a detailed overview of gut microbiota alterations in HD, as unraveled in clinical and preclinical studies (Table 1), the neuroimmunomodulatory role and influence of these microbes, and their secretory products in HD pathogenesis. We also discuss the microbiota–gut–brain axis in HD relative to other NDs and summarize therapeutic strategies to remodel the gut microbiota to delay disease onset and alleviate HD pathology safely and efficaciously.

**Table 1:** Summary of study characteristics and significant microbiota alterations in Huntington's disease. HC, healthy control; HD, Huntington's disease; HDGECs, Huntington's disease gene expansion carriers; PD, phylogenetic diversity; RCT, randomized clinical trial; WT, wild‐type.

Sample type, age, and sex/strain	Study site	Intervention	Microbiota alterations	Sex‐specific microbiota alterations	Microbial association with health measures	Method	Refs
**Clinical studies**							
42 HDGECs [22 males, 20 females], 36 HC [15 males, 21 females] 24–75 years Feces	Melbourne, Australia	NA	HDGEC compared to HC: Decrease in α‐diversity (observed species and Fisher's index) Significant differences in β‐diversity (unweighted UniFrac distances) Lower: Genus: *Eubacterium*	Males only Phyla: Euryarchaeota, Firmicutes, Verrucomicrobia Families: Acidaminococcaceae, Bifidobacteriaceae, Coriobacteriaceae, Erysipelotrichaceae, Methanobacteriaceae, Peptococcaceae, Peptostreptococcaceae and Rikenellaceae	Cognition, motor function, and disease progression Species: *Eubacterium hallii*	16S rRNA sequencing	[118]
33 HD [15 males 18 females], 33 HC [15 males 18 females] 42.6–48 years Feces	Beijing, China	NA	HD compared to control: increased α‐diversity (Chao 1, observed species, and Faith's PD) Significant differences in β‐diversity (unweighted UniFrac distances) Higher Phylum: Actinobacteria, class: Deltaproteobacteria Order: Desulfovibrionales Families: Oxalobacteraceae, Lactobacillaceae, Desulfovibrionaceae Genera: *Intestinimonas, Bilophila, Lactobacillus, Oscillibacter, Gemmiger*, and *Dialister* HC compared to HD: Higher Genus: *Clostridium XVIII*	NA	Total functional capacity Genus: *Intestinimonas* Cognition Genus: *Lactobacillus*	16S rRNA sequencing	[12]
*Postmortem* 7 HD [5 males, 2 females] No HC Brains			Higher bacterial genera: *Pseudomonas*, *Acinetobacter*, and *Burkholderia* Fungal genera: *Candida*, *Davidiella*, *Malassezia*, *Rhodotorula*, and *Ramularia*	NA	NA	16S rRNA and ITS1 sequencing	[121]
RCT 41 HDGECs [20 males, 21 females], 36 HC [15 males, 21 females] 24–75 years Feces	Melbourne, Australia	Probiotics *Lactobacillus rhamnosus*, *Saccharomyces cerevisiae* (*boulardii*), and *Bifidobacterium animalis* ssp *lactis*	NA	HC females only Significantly different only after treatment Family: Eggerthellaceae	Cognition, motor function, and disease progression: Species: *E. hallii*	16S rRNA sequencing	[119]
**Preclinical studies**							
R6/1 HD [6–8 males per group], WT [6–8 males per group] Feces	NA	High fiber diet	R6/1 HD mice compared to WT: No difference in α‐diversity (richness, Shannon) Significant differences in β‐diversity (Aitchison distance) Higher Phyla: Desulfobacterota Families: Bacteroidaceae, Butyricicoccaceae, Oscillospiraceae, Ruminococcaceae Lower Phyla: Actinobacteriota, Campylobacterota, Fusobacteriota and Proteobacteria Families: Campylobacteraceae, Carnobacteriaceae, Corynebacteraceae, Gemellaceae, Micrococcaceae, Neisseriaceae, Selenomonaceae, Weeksellaceae Order: Lactobacillales	NA	NA	16S rRNA sequencing	[240]
R6/1 HD [7 males, 11 females], WT [10 males, 7 females] Feces	NA	NA	R6/1 HD mice compared to WT: Higher Phylum: Bacteriodetes Order: Bacteroidales WT compared to R6/1 HD mice Higher Phylum: Firmicutes Order: Clostridiales	Higher in males only Phyla: Actinobacteria and Proteobacteria Lower in males Family: Deferribacteres	NA	16S rRNA sequencing	[130]
R6/1 HD [9 per sex], WT [9 per sex] Feces	NA	NA	R6/1 HD mice compared to WT: Significantly different Species: *Clostridium mt 5*, *Treponema phagedenis*, *Clostridium leptum CAG:27*, *Desulfatirhabdium butyrativorans*, *Plasmodium chabaudi*, *Defulfuribacillus alkaliarsenatis*, *Plasmodium yoelii* and *Chlamydia abortus*	NA	NA	Shotgun metagenomics	[31]
R6/2 HD [4–6 males], WT [4–6 males] Feces	NA	NA	R6/2 HD mice compared to WT: Higher Phylum: Bacteriodetes Significantly different Family: Enterobacteriaceae Genera: *Bacteroides*, *Parabacteroides*, *Lactobacillus* and *Coprobacillus* WT compared to R6/1 HD mice Higher Phylum: Firmicutes	NA	Blood glucose Genera: *Lactobacillus* and *Desulfovibrio* Body weight Family: Enterobacteriaceae Genus: *Parabacteroides* Increased intestinal permeability Phylum: Proteobacteria Genus: *Parabacteroides*	16S rRNA sequencing	[44]
R6/1 HD [9 males], WT [9 males] Feces	NA	NA	R6/1 HD mice compared to WT Increased α‐diversity (Shannon) Significant differences in β‐diversity (Aitchison distance) Higher *Penicillium solitum* and *Meyerozyma guilliermondii* Lower *Glarea lozoyensis*, *Malassezia restricta*, *Penicillium digitatum*, *Yarrowia lipolytica*, and *Aspergillus fischeri*, *Aspergillus uvarum*, and *Aspergillus alliaceus*	NA	NA	Shotgun metagenomics	[144]
R6/1 HD [7–8 per sex], WT [7–8 per sex] Feces	NA	Environment enrichment (EE) and exercise EE	NA	Males Significantly different in EE R6/1 HD compared to EE WT Order: Lachnospirales, Bacteroidales, Oscillospirales and Lactobacillales Significantly different in standard housing (SH) R6/1 HD compared to SH WT Order: Coriobacteriales, Bacteroidales, Monoglobales Significantly different in EX R6/1 HD compared to EX WT Order: Gastranaerophilales, Oscillospirales, Desulfovibrionales and Bacteroidales Females Significantly different in EE R6/1 HD compared to EE WT Order: Deferribacterales and Peptostreptococcales‐Tissierellales Bacteroidales and Lachnospirales	NA	16S rRNA sequencing	[38]

## Gut health and disease in HD

### GI disturbances in HD

Tight junctions (TJ) are formed by neighboring intestinal epithelial cells (IECs) and are essential for upholding the intestinal barrier and regulating the movement of substances such as water, ions, nutrients, and solutes across the intestinal epithelium [39]. These junctions are formed by an assembly of multiple proteins such as occludin, claudins, zonula occludens (ZOs), tricellulin, cingulin, and junctional adhesion molecules [40]. These proteins are pivotal to the maintenance of intestinal barrier integrity [39]. Alterations in the distribution of TJ proteins have been reported in PD patients and linked to increased intestinal permeability, with the latter correlating with the accumulation of α‐synuclein in the enteric nervous system (ENS) [41, 42]. Decreased expression of occludin, but not zonula occludens‐1 (ZO‐1), has been observed in colonic samples from PD patients [43]. Similarly, the gut barrier is compromised in HD, with HD presymptomatic gene carriers and patients showing signs of GI impairment and bowel abnormalities comparable to inflammatory bowel disease (IBD). Fatal weight loss, a devastating manifestation seen in HD, has been linked with the GI dysfunction described above [13].

**Fig. 1:**
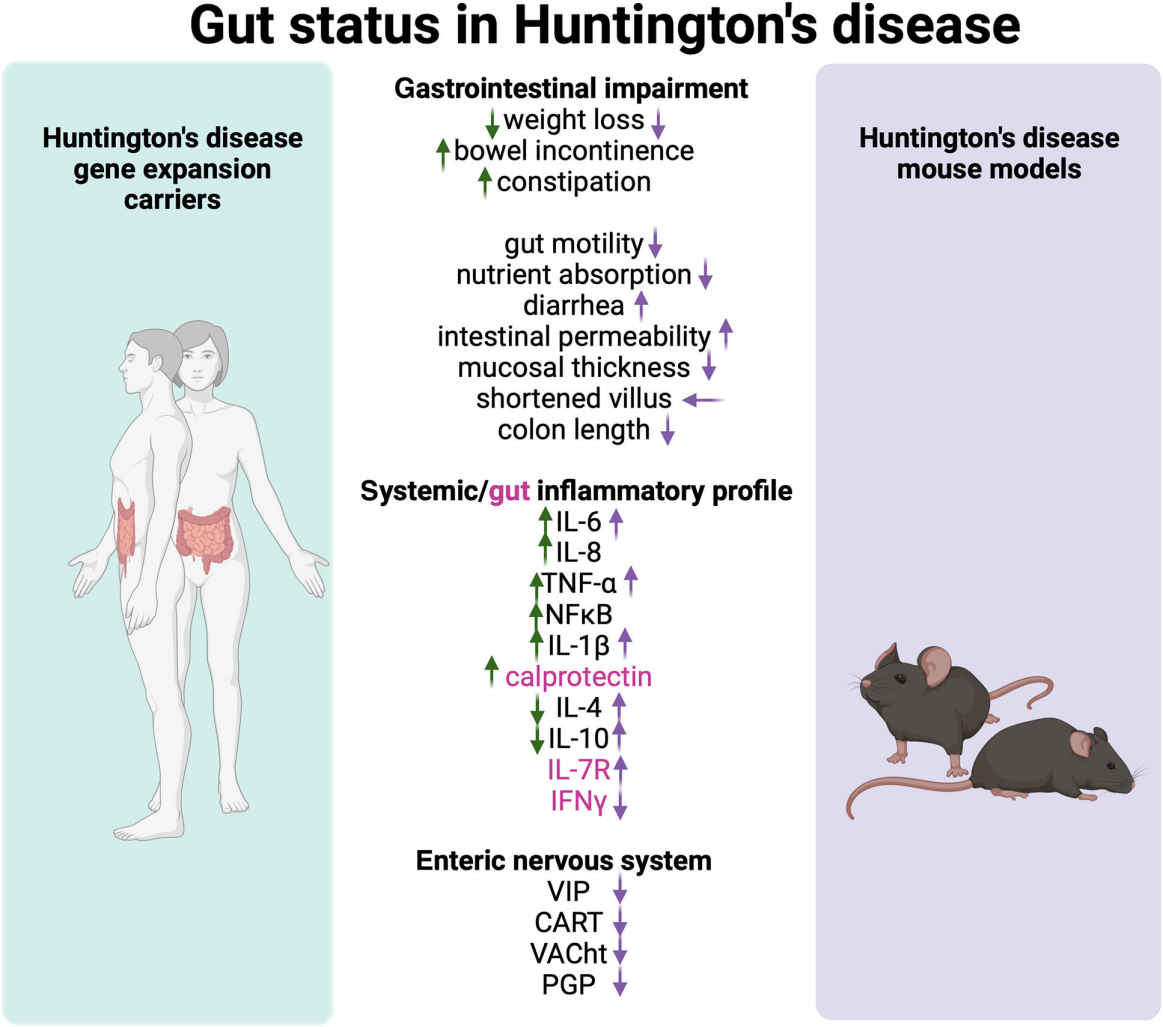
Schematic illustration of the current known gut status and immune dysregulation in Huntington's disease subjects and animal models. Abnormalities in the gastrointestinal tract of Huntington's disease subjects and mice as well as the decreased expression of enteric neuropeptides. Immune dysregulation is also depicted, with differential levels of pro‐inflammatory and anti‐inflammatory cytokines in Huntington's disease subjects and mouse models. ↑: increase, ↓: decrease, ←: promotes. Green arrows represent clinical findings from human studies and purple arrows represent preclinical findings from animal studies. Created with BioRender.com.

Decreased body weight, impaired gut motility, nutrient malabsorption, decreased mucosal thickness, shortened villi length and diarrhea have been reported in HD mouse models [13, 44]. The R6/1 and R6/2 HD mice are models of adult‐onset and juvenile‐onset HD respectively [45, 46] and have been described in detail in Box 1. Increased intestinal epithelial permeability is interchangeably described as ‘leaky gut’. Studies have shown that in the early stage (12–14 weeks) in the R6/1 HD model, there is no difference in intestinal permeability and gut macroscopy (with the latter characterized as colon length, cecal weight, and length), between HD and wild‐type (WT) littermates [1, 38]. However, differences are visibly detected at the late disease stage (20 weeks) in both male and female HD mice. Decreased colon length in these mice was also reported [1, 38]. In the same vein, R6/2 HD mice showed increased intestinal permeability and decreased colon length at 16 weeks of age [44] (Fig. 1).

Box 1 **Animal models of HD**

The R6/1 and R6/2 lines were developed in 1996 and were the first transgenic mouse models of HD, with each solely expressing exon 1 of the human *huntingtin* (*HTT*) gene with 115 and 120–150 CAG repeats respectively thus producing 31% and 75% of endogenous HTT protein [45, 46]. The transgene (derived from a HD patient) expression in these mice is driven by the human *HTT* promoter and although both lines exhibit HD‐like deficits and pathophysiology, the R6/2 mouse develops disease phenotype most rapidly and a marked accumulation of HTT aggregates in the brain [45, 311]. The bacterial artificial chromosome (BAC) transgenic mouse model of HD (BACHD) expresses full‐length human mutant huntingtin with 97 glutamine repeats and exhibits progressive motor deficits, neuronal synaptic and psychiatric anomalies as well as late‐onset selective cortical and striatal atrophies [312, 313]. Yeast artificial chromosome (YAC)128 is a transgenic mouse model of HD expressing a human mHTT gene containing 128 CAG repeats while the YAC18 control mouse line possesses 18 CAG repeats in the human HTT transgene [62, 68]. The OVT73 sheep model of HD has the full‐length human *HTT* cDNA transgene and a total of 73 CAG repeats and represents an early prodromal disease stage [314]. These and other models of HD have been extensively reviewed elsewhere [315].

### Gut barrier integrity

Gut barrier integrity is vital in maintaining intestinal and host health. In healthy individuals, the gut acts as a barrier, preventing the movement of bacteria from the intestinal lumen into the systemic circulation [44]. The mucus secreted by the gut layers and the tight junctions between epithelial cells, consisting of TJ proteins such as zonula occludens (e.g., ZO‐1), claudins, and other junction adhesion molecules, obstructs pathogen entrance and maintains tissue homeostasis [47, 48].

The gut microbiota and their secretory products can modulate the integrity of the blood–brain barrier (BBB); thus, BBB dysfunction has been suggested as an early biomarker of neurodegeneration [49]. Increased BBB leakage is evident in Alzheimer's disease (AD) and PD patients as well as in mouse models of these diseases [49, 50]. Similarly, the BBB is disrupted in HD and evidence of decreased expression of BBB TJ proteins in R6/2 HD mice has been presented [51, 52]. However, this was not corroborated by Stan *et al*. [44], as they did not observe significant differences in the expression of occludin and ZO‐1 levels in the cortex and colon at 12 and 16 weeks of age between R6/2 HD mice and WT littermates. At 18 weeks of age, a very late stage in this juvenile‐onset mouse model of HD, Stan *et al*. [44] did observe a decreased expression of occludin in the colon mucosa of R6/2 HD mice, as well as disruptive epithelial remodeling. This disparity of findings, despite using the same HD model (R6/2 transgenic mice), could be attributed to CAG repeat size (which can vary within the same transgene across different mouse colonies), and other genetic, environmental and experimental variables. Importantly, the R6/2 HD mice in Stan *et al*.'s study had a CAG repeat size ranging between 242 and 257 instead of 120–150 as described in Box 1. A previous study had reported that further increases in CAG repeat size (into the extreme range, beyond 150 repeats) can paradoxically prolong survival, and delay the onset of disease and neurodegeneration in the R6/2 HD mice [53].

### The enteric nervous system in HD

The enteric nervous system (ENS) is in the wall of the GIT and is composed of neuronal and glial cells, and associated neural circuitry, that is crucial for the regulation of diverse GI functions [54]. Enteric neuropeptides consist of small chains of amino acids and are an important class of signaling biomolecules produced by neurons and other cell types in the brain and the gut and regulate immune activities in the gut as well as bidirectional communication between the brain and the gut [55]. Notably, enteric neuropeptides can exert excitatory or inhibitory influences on gut motility [56]. The role of gut innervation and communication between the ENS and central nervous system (CNS) in NDs has been extensively reviewed [57] and possible ways through which the gut microbiota and their derived components and metabolites influence the CNS and ENS in neurological disorders have been summarized [58]. However, it is pertinent to highlight that mHTT is expressed ubiquitously in the body, not just the CNS, and mHTT protein notably aggregates in the GIT (stomach, duodenum and rectum and the ENS), as seen in mouse models of HD [13].

In addition to mHTT‐immunoreactive inclusions (aggregates), decreased expression of the enteric neuropeptides such as vasoactive intestinal polypeptide (VIP), cocaine‐ and amphetamine‐regulated transcript (CART), protein gene product (PGP) and vesicular acetylcholine transporter (VaCHT) have been reported in R6/2 HD mice [13]. Notably, these findings were supported in a case study of a 56‐year‐old woman with mild HD (40 CAG repeats in the HTT, which is usually associated with later onset) who not only showed decreased expression of these neuropeptides but also morphological abnormalities in the villi [59]. Taken together, the altered expression of enteric neuropeptides and the presence of mHTT aggregates in the GIT, as well as associated morphological abnormalities, highlight the significant role of the ENS and the need for a better understanding of the interactions between gut microbes and the ENS in HD.

## Inflammatory responses in HD

The mammalian gut is the largest immune organ and consists not only of the gut microbes but of gut epithelia, goblet cells, and Paneth cells as well as macrophages, dendritic cells, and T‐cells that are responsible for maintaining barrier integrity and immune cell homeostasis [60, 61]. Alterations in immune profiles in HD subjects are known and there is evidence of innate immune activation in premanifest and manifest HD gene carriers, thus immune dysfunction has been implicated in the pathogenesis of HD [62, 63, 64, 65, 66, 67]. The HTT gene mutation is expressed by immune cells from HD patients and this may contribute directly, or indirectly, to changes in inflammatory cytokines [65]. Additionally, microglia, monocytes, and macrophages from HD patients are hyper‐reactive when stimulated by lipopolysaccharide (LPS). This hyperactivity was also reproduced in the YAC128 mouse model of HD (but not the control YAC18 mice), which suggests a crucial role of immune cell derangement in HD pathogenesis [62, 68].

Increased levels of the proinflammatory cytokines TNF‐α, IL‐6, and IL‐8 were observed in the plasma of HD patients, while the anti‐inflammatory cytokines IL‐4 and IL‐10 increased significantly with disease progression [62]. IL‐8 positively correlated with worsening disease symptoms and negatively with Total Functional Capacity (TFC), a measure of functional independence. Interestingly, increased plasma levels of IL‐6 were observed in premanifest individuals about 16 years before the onset of motor symptoms [62].

However, in another study, decreased plasma levels of IL‐4 were seen in HD, but no significant differences in IFN‐γ, IL‐1β, IL‐2, IL‐6, IL‐8, IL‐10, IL‐12p70, IL‐13, or TNF‐α were reported [12]. Importantly, the genus *Intestinimonas* was positively correlated with IL‐4 while *Bilophilia* was negatively associated with IL‐6 [12]. Additional evidence of chronic immune activation in HD patients and animal models as well as clinical trial findings of immune‐based therapies for HD have been extensively reviewed elsewhere and are beyond the scope of this review [69]. NFκB, which is involved in immune cell regulation, is upregulated by mHTT [70] and consequently upregulates IL‐6 and IL‐8 [71, 72]. Furthermore, its inflammatory cascade can be suppressed by gut bacteria such as *Faecalibacterium prausnitzii* and select strains of *Clostridium* [73].

Recently, a systematic review and meta‐analysis of 10 human studies assessing peripheral markers of inflammation in HD revealed elevated plasma levels of IL‐6 and IL‐10 in HD subjects compared to healthy controls [74]. Although C‐reactive protein (CRP) was also elevated in the HD group, it was non‐significant [74]. Notably, there were no differences in the plasma/serum levels of these biomarkers among premanifest and manifest subjects [74].

Comparably, evidence of intestinal inflammation has been presented and linked with the occurrence and progression of PD. Immunologic profiling of colonic biopsies obtained from PD patients (*n* = 14) and matched healthy controls (*n* = 14) showed increased mRNA expression of TNF‐α, IFN‐λ, IL‐1β, and IL‐6 as well as the glial markers, glial fibrillary acidic protein (GFAP) and Sox‐10, particularly in the ascending colon of PD patients [75]. Additionally, the levels of these differentially expressed mRNAs were negatively correlated with disease duration. Stool immune profiling showed elevated levels of IL‐1α, IL‐1β, CXCL8 and CRP in PD patients (*n* = 156) relative to healthy controls (*n* = 110) but was not correlated with subject age or disease duration [76].

Calprotectin is a robust biomarker of GI inflammation and a non‐invasive approach to diagnosing and assessing Crohn's disease and ulcerative colitis [77]. Increased levels of fecal and serum calprotectin are known in PD [78, 79, 80]. Elevated plasma levels of calprotectin were uncovered in moderate HD patients compared to healthy controls [81]. More evidence linking intestinal inflammation to NDs has been summarized elsewhere [57, 82].

Like HD patients, heightened inflammatory responses have been shown in experimental models of HD and are extensively summarized here [83]. Notably, elevated levels of serum IL‐6, IL‐10, IL‐1β and IL12p70 and plasma IL‐6, TGF‐β1 and matrix metalloproteinase (MMP)‐9 were reported in the R6/2 HD mice [62, 84]. Male R6/1 HD mice showed higher levels of IL‐7R in the proximal colon while females showed a decrease in IFN‐γ [1]. Taken together, these studies suggest a link between systemic inflammation and gut microbes in HD and open an opportunity to investigate the therapeutic potential of live biotherapeutics for targeted immunomodulation.

## The vagus nerve in the microbiota–gut–brain axis

The autonomic nervous system is a component of the peripheral nervous system that regulates involuntary physiological processes and activities such as breathing, heart rate, blood pressure digestion and sexual arousal [85]. It consists of three distinct divisions which are the parasympathetic, sympathetic and enteric nervous systems [85]. The vagus nerve is the 10th cranial nerve and a crucial component of the parasympathetic nervous system [86]. It is a mixed nerve fiber composed of approximately 80% afferent and 20% efferent fibers [87].

The vagus nerve as the link between the CNS and the ENS, and the way in which the gut microbiota hijacks it to communicate with the brain, have been extensively described elsewhere [35, 88, 89]. Briefly, gut microbes can produce metabolites and neuroactive molecules such as γ‐aminobutyric acid (GABA), serotonin, dopamine, and acetylcholine, that send signals between the afferent neurons in the ENS and the brain via the vagus nerve [90]. Cholecystokinin (CCK) regulates GIT function by inhibiting gastric emptying and food intake through the activation of CCK‐1 receptors on the vagal afferent fibers innervating the GIT [90]. Short‐chain fatty acids such as butyrate can directly affect vagal afferent terminals while long‐chain fatty acids such as oleate can activate vagal afferent fibers by a CCK‐dependent mechanism [91].

The vagus nerve has immunomodulatory properties. Importantly, a cholinergic anti‐inflammatory pathway (CAIP) is mediated through the release of acetylcholine by the efferent vagus nerve fibers [91]. Once activated, CAIP can dampen peripheral inflammation by suppressing the synthesis and release of TNF‐α, decrease intestinal permeability by reinforcing tight junctions, and possibly alter microbiota composition [91]. This has been extensively discussed elsewhere [91, 92]. Enteroendocrine cells (EECs) constitute 1% of the IECs and release their content in the presence of nutrients such as carbohydrates, proteins, and triglycerides, modulating gut functions such as motility, secretion, and food intake [93]. EECs interact with the vagal afferents either directly through the release of serotonin or gut hormones such as CCK, glucagon‐like peptide 1 (GLP‐1), and peptide YY [93]. Toll‐like receptors (TLRs) recognize bacterial products such as LPS and are expressed by EECs. TLR4s are expressed on the vagal afferent fibers [94], and these fibers can sense bacterial products and activate the brain [91].

There is evidence of low modulation of autonomic cardiovagal activity in HD patients as characterized by a reduction in heart rate variability at rest and during deep respiration, suggesting autonomic dysfunction [95] and this seems to occur approximately 20 years before the onset of motor deficits [96]. Stress inhibits the vagal nerve [91]. Vagal tone, which describes the level of vagal activity is correlated with stress response regulating capacity [88]. A low vagal tone is evidenced in patients with IBD and irritable bowel syndrome (IBS), thus permitting peripheral inflammation [97, 98]. Treatments that target the vagal nerve increase vagal tone and decrease cytokine production [88].

Major depressive disorder (MDD) is often associated with NDs such as HD, PD and AD and subjects may not effectively respond to currently approved antidepressants [99]. Importantly, 15–69% of HD patients suffer from comorbid MDD [99], thus suicide is a leading cause of death in HD patients compared to the general population [100]. Monoamine neurotransmitters such as serotonin (5‐hydroxytryptamine), norepinephrine and dopamine regulate emotions and can influence MDD [101]. Stimulating the vagal afferent fibers in the gut influences the monoaminergic brain system, which plays a crucial role in major psychiatric conditions such as mood and anxiety disorders [88]. Gut bacteria may have a beneficial effect on mood and anxiety, partly by influencing vagal activity [88, 102, 103]. However, vagotomy studies have also shown that the gut microbiota and their secretory products may promote depressive behavior in rodents in a vagal nerve‐dependent way [104, 105, 106].

Vagal nerve stimulation (VNS) entails implanting a small device to send electrical impulses to the vagus nerve [107]. Consistent evidence suggests that VNS has promising potential as an add‐on treatment for IBD [108], PD [109], treatment‐resistant depression [110] and AD [111] but has not been investigated in HD yet. Moreover, our knowledge of the microbiota‐vagus‐brain interactions in HD is limited and the dearth of literature in this essential area justifies the need for properly designed studies in preclinical models of HD and potentially in patients.

## Gut microbiome in HD

The most dominant bacterial phyla among the intestinal microbiota are Bacteroides and Firmicutes. Their abundance may be inversely altered in disease states. Thus, the ratio of these phyla has been indicated as a biomarker of gut health and stability and correlates with various diseases, including obesity and inflammatory bowel syndrome [112, 113]. A change in this ratio has also been linked to aging and, consequently, neurodegeneration [114, 115]. Microbial profiling of 1550 healthy participants from the Ukrainian population showed that, in both sexes, the Firmicutes‐to‐Bacteroides (F/B) ratio increases with age [116]. Similarly, in healthy sexually mature male Sprague–Dawley rats, the relative abundance of Firmicutes increased with age, but Bacteroides declined. This ratio is also positively associated with BMI [116] and body weight [112]. However, variable F/B ratios have also been reported in healthy individuals and healthy preclinical models in other studies, raising questions about its reliability as an indicator of normal intestinal homeostasis [115, 117]. Moreover, there is evidence that microbial richness increases with age, while the reverse is the case with bacterial diversity [115]. Thus, there is a compelling need to understand the gut microbiota signatures in HD subjects and experimental models of HD relative to their healthy counterparts.

### The gut microbiota of patients with HD

The gut microbiome of HD patients has not been extensively studied compared to other NDs such as AD, PD, amyotrophic lateral sclerosis (ALS) and multiple sclerosis (MS). Altered microbial profiles in HD gene expansion carriers (HDGECs – a combination of premanifest and manifest subjects; *n* = 42) compared to healthy controls (*n* = 36) have been shown [118, 119]. While there was no difference in microbial profiles between premanifest and manifest, decreased α‐diversity and differences in β‐diversity was reported in the HDGEC group, compared to controls and these findings were reproduced in a subsequent study from the same group [118, 119]. Furthermore, a lower abundance of the common gut microbe *Eubacterium hallii* was negatively correlated with severe motor symptoms in manifest HD patients as well as with proximity to disease onset in premanifest individuals. Contrarily, *E. hallii* was positively associated with cognitive function [118].

Intriguingly, microbial profiling of fecal samples from a different population (participants from China; as opposed to participants studied by Wasser *et al*., who were from Australia) revealed a significant increase in species richness (α‐diversity) and differences in microbial structure (β‐diversity) in HD patients (*n* = 33) compared to controls (*n* = 33), as well as altered relative abundance of different taxa [12]. Specifically, an increased abundance of the phylum Actinobacteria, the class Deltaproteobacteria, the order Desulfovibrionales, the families Oxalobacteraceae, Lactobacillaceae, Desulfovibrionaceae, and the genera *Intestinimonas*, *Bilophila*, *Lactobacillus*, *Oscillibacter*, *Gemmiger*, and *Dialister*, were seen in HD patients compared to controls, whereas the genus *Clostridium XVIII* was significantly elevated in healthy controls compared to HD patients. Importantly, the butyrogenic bacteria *Intestinimonas* was positively correlated with TFC, while *Lactobacillus* was negatively associated with cognitive outcomes as assessed by the Mini‐Mental State Examination (MMSE) score [12]. The disparity in findings between these clinical studies may be attributed to factors such as ethnicity, geography, host genetics, age, and sample size [12].

**Fig. 2:**
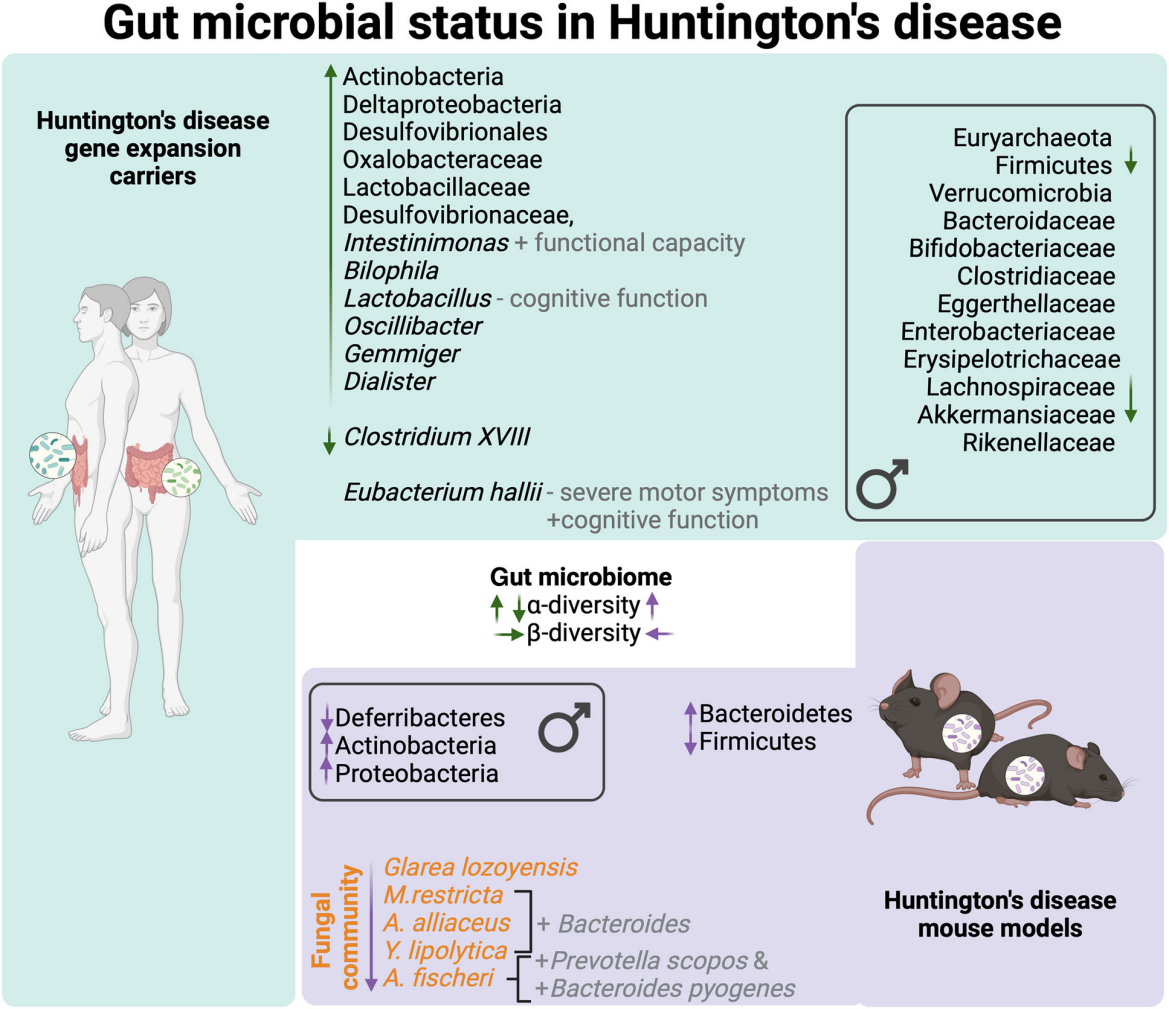
Schematic illustration of the current known gut microbial status in Huntington's disease subjects and animal models. Variations in gut microbial composition in Huntington's disease subjects and mice are known. The known connections between altered gut bacteria and their associations with neurological function in the context of Huntington's disease are shown. Additionally, bacterial‐fungal interactions and sex‐specific patterns within the gut microbiota are highlighted. ↑: increase, ↓: decrease, ←: promotes, +: positive correlation, −: negative correlation. Green arrows represent findings from human studies and purple arrows represent findings from animal studies. Created with BioRender.com.

Sex‐specific differences in HD microbiota have been reported in HD patients [118]. Wasser *et al*. observed significant differences in the abundance of the phyla Euryarchaeota, Firmicutes, Verrucomicrobia, and the families Bacteroidaceae, Bifidobacteriaceae, Clostridiaceae, Eggerthellaceae, Enterobacteriaceae, Erysipelotrichaceae, Lachnospiraceae, Rikenellaceae among others in male HD subjects compared to controls, but not in females [118] (Fig. 2). This is in line with evidence from other studies showing sex‐specific differences in gut microbiota composition in NDs as extensively reviewed elsewhere [120].

Like fungal infections (discussed later), evidence for bacterial DNA in cerebrospinal fluid (CSF) and neural tissues in, HD, PD, and AD and ALS has been demonstrated [121, 122, 123, 124, 125, 126, 127, 128]. In HD brains, a high abundance of bacterial genera including *Pseudomonas*, *Acinetobacter*, and *Burkholderia* was reported [121]. However, a main caveat of this study was that post‐mortem samples from HD patients (*n* = 7), but no control subjects were assessed, although they referenced a previous study that included controls [121]. Additionally, the sequencing runs lacked appropriate controls, and the possibility of post‐mortem contamination calls for a cautious interpretation [121].

Furthermore, amyloid deposits have been reported in the brains of ALS, AD, PD, and HD patients [129]. Amyloid peptides, known for their antimicrobial/antifungal activity, can be triggered by microbial infections, giving rise to an innate immune response. Corpora amylacea (CA) are amyloid bodies reportedly found in the striatum of HD patients and have also been observed in other NDs [124]. Although fungal proteins in CA were identified in CNS samples of AD, PD, and ALS patients and reacted to fungal antibodies, CA from AD patients seemed to be more abundant [122, 124, 125, 128].

### The gut microbiota in murine models of HD

There is consistent evidence of altered microbial composition in the R6/1 HD model, especially at 12 weeks of age [1, 31, 38, 130]. 16S rRNA sequencing analysis revealed an elevated abundance of Bacteroidetes and a proportional decrease in Firmicutes, while the reverse was seen in WT [130]. Specifically, microbiome signatures in WT mice primarily consisted of Clostridiales from the Firmicutes phyla while HD microbiome signatures mostly consisted of Bacteroidales from the Bacteroiodetes phyla [130]. Further analysis revealed increased microbial diversity in HD males and reported no similar observation in females. Deferribacteres were significantly lower in HD males, while Actinobacteria and Proteobacteria were significantly higher in HD males compared to male WT littermate controls; no such differences in the trio were observed in females [130]. These preclinical discoveries in HD mice are consistent with sex differences in gut microbiota subsequently described in HD clinical studies [118, 119].

Interestingly, shot‐gun sequencing metagenomics revealed 30 genotype‐specific microbiome signatures at 12 weeks of age in R6/1 HD mice relative to WT littermate controls [31]. These signatures include *Clostridium mt 5*, *Treponema phagedenis*, *Clostridium leptum CAG:27*, *Desulfatirhabdium butyrativorans*, *Plasmodium chabaudi*, *Defulfuribacillus alkaliarsenatis*, *Plasmodium yoelii* and *Chlamydia abortus* [31]. Notably, examination of microbial genes and KEGG orthology (KO) pathway signatures at week 12 showed a key difference in decreased levels in galactose metabolism and benzoate degradation and increased levels in sulfur metabolism, lysine degradation, glutathione metabolism and butanoate metabolism in HD microbiome relative to WT [31].

The microbiome is shaped by stability and volatility with the latter being described as an alteration in microbiota composition over time. Gut microbiome stability is crucial for host health and volatility has been associated with disease states such as IBD and stress [131, 132]. Kong *et al*. [31] uncovered heightened volatility in HD mice compared to the stability seen in WT littermates and highlighted fatty acid metabolism, fatty acid biosynthesis, tryptophan metabolism, and propanoate metabolism as some of the pathways mainly influenced by volatility in the HD microbiome.

Similarly, evidence of gut dysbiosis has been presented in R6/2 HD mice at 16 weeks of age with increased relative abundance of Bacteroidetes and decreased Firmicutes in these mice relative to WT and is consistent with what was seen previously in R6/1 HD mice [44]. Although there were no differences in α‐diversity, microbiota composition was significantly different between both genotypes. Bacteria significantly and distinctly associated with the R6/2 HD mice were *Bacteroides*, *Parabacteroides*, *Lactobacillus*, *Coprobacillus*, and the Enterobacteriaceae. Like HD subjects, the R6/2 HD mice are prone to have elevated blood glucose and this study uncovered a positive correlation between blood glucose and *Lactobacillus* and a negative correlation with *Desulfovibrio*. Additionally, body weight was negatively associated with Enterobacteriaceae and *Parabacteroides* while increased intestinal permeability was positively linked to the abundance of gram‐positive bacteria, notably the Proteobacteria and *Parabacteroides* phylum and genus respectively [44].

### The gut mycobiota in HD

Over 400 fungal species in humans have been identified [133] with fungal communities accounting for 0.1% of fecal microbial DNA. The distal colon is the most colonized portion of the intestine with respect to fungal communities [134]. Human and murine mycobiomes are mainly dominated by the phyla Ascomycota and Basidiomycota. Although the influence of the gut mycobiome in neurological disorders has been reviewed [135], it is pertinent to highlight that high levels of fungal antigens as well as fungal polysaccharides were detected in AD patients through a multi‐omics approach [123]. *Malassezia* spp., *Phoma*, and *Saccharomyces cerevisiae* were identified in the CNS of AD patients [136]. Alonso *et al*. further showed the presence of fungal species in various brain regions of AD patients but none in healthy controls, suggesting fungal infections [123, 124, 125]. Similar observations were seen in ALS and PD subjects. The genera *Candida*, *Malassezia*, *Fusarium*, *Botrytis*, *Trichoderma* and *Cryptococcus* were more prominent in the neural tissues of ALS patients relative to the control neural tissues [122]. Intriguingly, fungal structures were detected in brain sections of HD patients and following genomic sequencing uncovered fungal genera (*Candida*, *Davidiella*, *Malassezia*, *Rhodotorula*, and *Ramularia*) which were similarly observed in other NDs except *Ramularia*, which appeared to be an HD‐specific genus [121].

The evidence provided suggests that opportunistic fungal species such as *Candida albicans* can translocate from the gut to the brain and trigger an inflammatory response, as shown in monocolonized germ‐free (GF) mice [137]. Although aging has been described as a contributing factor to gut dysbiosis and may increase the possibility of such migration, there was no evidence of fungal dysbiosis in aged specific pathogen‐free (SPF) mice (24 months) [137]. Moreover, the relative abundance of fungal species in older and younger SPF mice showed no significant difference [137] and depletion of gut bacteria communities (using broad‐spectrum antibiotics) rapidly expanded caecal colonization by *Ca. albicans* in the mice [137].

Commensal fungi play an indispensable role in host health and immunity. Fungi such as *Yarrowia lipolytica* have been demonstrated as probiotics [138]. Importantly, gut fungi can produce neurotransmitters and other metabolites that exert a neuroimmunomodulatory role [134, 139, 140]. However, the mycobiome has been implicated in diverse diseases such as gastric cancer, colorectal cancer, and inflammatory bowel disease, and recently Alam *et al*. showed that the fungal microbiome upregulated IL‐33 secretion in pancreatic cells and consequently drove type 2 immunity [141] and *Ca. albicans* induced the expression of IL‐35 in M2 macrophages [142]. Importantly, disruptions in the bacterial‐fungal relationship have been linked to neurological disorders such as autism spectrum disorder (ASD), AD, and PD [143].

While the fecal bacterial phyla and families in HD have been characterized by a few studies, the fungal gut residents have received unequal attention. Significant alterations of the intestinal fungal community in the R6/1 HD mouse model compared to controls, specifically at 12 weeks of age, which is before the onset of motor deficits, have been shown [144]. Not only was there a significant difference in β‐diversity in HD mice as well as increased α‐diversity compared to WT, but the authors also uncovered a signature of 15 fungal species driving the major compositional differences between WT and HD. Further analysis revealed bacteriome‐mycobiome interactions in this HD model and a strong positive association of HD‐depleted fungal species *Glarea lozoyensis*, *Malassezia restricta*, *Aspergillus alliaceus*, and *Y. lipolytica* with the genera *Bacteroides*, and a positive association of *Aspergillus fischeri* solely with *Prevotella scopos* and *Bacteroides pyogenes*. Intriguingly, *Lactobacillus reuteri* was negatively associated with *G. lozoyensis*, *M. restricta*, *A. alliaceus*, and *Y. lipolytica* [144] (Fig. 2).

A decrease in fungal richness was observed in the 3xTg AD mouse model compared to WT, though not significantly. Importantly, a significant increase in the fungal Dipodascaceae family was reported [145] and in WT mice an abundance of Basidiomycota and Ascomycota was observed, compared to the AD mice.

Chitin, a polysaccharide component of fungal cell walls, is a precursor for chitinase, the latter highly recognized as a biomarker for HD with elevated levels reported in CSF [146]. This enzyme is produced by astrocytes and macrophages [147, 148]. Chitin has also been reported in AD samples from the CNS. Additionally, fungal proteins, enolase, and β‐tubulin were uncovered in the CA from AD patients [125].

Overall, the gut mycobiome signature in HD subjects requires more characterization which would improve our understanding of trans‐kingdom network interactions and how they influence immune activity and the onset and progress of HD. Furthermore, we need to understand how fungal‐altering approaches can influence bacterial communities, as this may have therapeutic implications.

## Gut microbiota‐derived metabolites in HD

Gut microbiota‐derived metabolites are effectors of crosstalk between the gut and the brain and have been suggested as credible biomarkers [149]. These biomolecules modulate neuronal function and influence diverse pathways in age‐related NDs [120]. Here we discuss a few of them and their influence on HD pathophysiology.

### Bile acids

Bile acids (BAs) are terminal derivatives of cholesterol metabolism and can influence immune homeostasis in hosts. Primary bile acids such as cholic acid (CA) and chenodeoxycholic acid (CDCA) undergo modification by intestinal microbiota, transforming them into secondary BAs which are transported to the liver through the enterohepatic circulation, or systemically [150, 151].

Bile acids regulate intestinal epithelial integrity as well as innate immune responses. Secondary BAs promote autophagy, especially the activation of the host's TGR5 receptor [152, 153]. Conversely, primary BAs inhibit autophagy by binding to the host's Farnesoid X receptor (FXR) on myeloid cells, thus suppressing autophagy, and proinflammatory responses, and promoting gut intestinal epithelial barrier function [153, 154].

There is a reciprocal interaction between BAs and the intestinal microbiota. BAs can directly exert antimicrobial actions or indirectly modulate gut resident communities through FXR‐induced antimicrobial peptides [153, 155, 156]. Conversely, gut microbes influence BA metabolism. Notably, a low abundance of BAs in the intestinal lumen upregulates LPS‐producing gram‐negative bacteria such as Bacteroides, while the reverse supports the abundance of gram‐positive microbes such as Firmicutes [155, 157].

Antibiotic treatment inhibits the expression of CYP7A1, an enzyme involved in cholesterol breakdown and bile acid synthesis [158, 159]. Differential expression of this gene has also been reported in GF mice [160]. These lines of evidence buttress the critical role of the gut microbiome in maintaining bile acids and their metabolic pathways.

Furthermore, BAs have been identified in the brains of rats [161]. Keene *et al*. [162] showed that systemically administered bile acids can reach the brain and exert a neuroprotective role. Conversely, the Baloni *et al*. [163] study showed that BAs synthesis pathway genes are expressed in the brains of AD patients and reported an association between BAs and cognitive deficits.

Tauroursodeoxycholic acid (TUDCA) is an endogenous hydrophilic bile acid synthesized by the conjugation of the secondary bile acid ursodeoxycholic acid (UDCA) with taurine. TUDCA is well tolerated and produced at low levels in humans. UDCA is an FDA‐approved medication for treating cholestasis and Ursodiol, a commercial form of UDCA, has been tested in clinical trials for the treatment of HD [164, 165].

Huntington's disease is associated with neuronal mitochondrial perturbations [166]. There is strong evidence that TUDCA can stabilize mitochondrial membrane potential, decrease reactive oxygen species (ROS) production, and attenuate apoptotic pathways in HD [162]. TUDCA significantly decreased 3‐nitro propionic acid (3‐NP)‐mediated striatal neuronal apoptosis in studies using *in vivo* models of neuronal excitotoxicity [162, 167]. TUDCA‐treated R6/2 HD mice showed minimized striatal degeneration and improved sensorimotor and cognitive phenotypes, as characterized by the rotarod and open field tests, respectively [168].

Overall, the neuroprotective role of these BAs and their precursor UDCA has been demonstrated in humans and experimental models of diseases, including HD, AD, PD, MS, and ALS, and has been extensively reviewed with ongoing clinical trials detailed here [164, 165, 169, 170, 171, 172].

### Purine metabolites

Host nucleotide metabolism is greatly influenced by the gut microbiota [173, 174]. For example, in fruit flies, *Lactobacillus plantarum* decreased dietary purine metabolites, *Lactobacillus brevis* and *Lactobacillus murinus* specifically decreased dietary adenosine, whereas *Acetobacter persici* increased allantoin, an end‐product of purine metabolism, in aged flies [173]. Allantoin has been shown to improve neurogenesis in the hippocampus as well as enhance cognition and memory in normal naïve mice [175].

Bacteroides may promote urate conversion to allantoin and thus influence serum uric acid levels in humans [176]. Xanthine oxidase is a secretory product of the gut microbiota and is important for the oxidative metabolism of purines [177]. Impaired purine metabolism and signaling have been implicated in the pathogenesis of HD. Post‐mortem brain tissues of HD subjects showed markedly increased urea and this was equally seen in a transgenic prodromal sheep model of HD, OVT73 [178]. Additionally, elevated plasma and CSF levels of inosine, hypoxanthine, xanthine, uric acid, and uridine have been reported in both HD mouse models and HD patients [179, 180] whilst decreased plasma levels of adenosine triphosphate (ATP) and pipecolic acid were negatively associated with Bacteroides in R6/1 HD mice [31].

Early‐onset HD patients show a cognitive decline which may eventually result in dementia [181]. Purinergic receptors have been associated with cognitive disturbances in NDs [182]. Notably, increased expression of P2X7R mRNA and protein have been unraveled in the cortical and striatal neurons of transgenic mouse models of HD and the administration of P2X7R antagonists has been shown to improve motor deficits and prevent body weight loss in R6/1 HD mice [183, 184]. Similarly, administration of the adenosine receptor A2AR antagonist istradefylline rescued working memory defects and long‐term depression anomalies in the cortico‐striatal synapse [185]. There is a strong positive association of formate, mannose, and xanthine with superior cognition [186]. Lower levels of xanthine, hypoxanthine, and adenosine have been observed in the frontal cortex of AD patients [187]. Moreover, transcriptional signatures of R6/2 HD mice showed an upregulation of *Pnp* and *Xdm*, both involved in purine metabolism [188]. Thus, modulating purinergic systems has been highlighted as a suitable therapeutic strategy for HD [179, 189].

### Branched‐chain amino acids and branched‐chain fatty acids

Although the diet is a chief source of branched‐chain amino acids (BCAAs), gut microbiota are actively involved in the *de novo* synthesis and uptake of amino acids [190]. BCAAs are important for the synthesis of neurotransmitters involved in memory and learning, such as acetylcholine, glutamate, and GABA [191]. Plasma levels of these neurotransmitters have been negatively linked with dementia [192, 193]. Decreased BCAAs such as isoleucine, leucine, and valine have been identified as potential biomarkers in HD models, including presymptomatic HD sheep (*OVT73*) [194] and evidence of an association between BCAA levels with CAG repeat length and disease progression has been shown [195, 196]. In contrast, the Castilhos *et al*. study [197] does not support this, and Andersen reported enhanced cerebral BCAA metabolism, especially isoleucine in the R6/2 HD model [198].

Nonetheless, alterations in BCAA levels have been implicated in metabolic disorders and NDs including AD, PD and ALS and have been extensively discussed [199, 200]. Decreased plasma and fecal levels of aromatic amino acids (AAAs) and BCAAs in PD patients have been reported as well as a decreased expression of *ilvB*, *ilvC*, *ilvD*, and *ilvN*, genes involved in BCAA biosynthesis pathways [201]. Interestingly, the altered AAA and BCAA levels were significantly correlated with microbial taxa Desulfovibrionaceae, Acidaminococcaceae, and Erysipelotrichaceae, while Streptococcaceae, *Streptococcus*, and *Lactobacillus* shared a negative association [201].

Branched‐chain fatty acids (BCFAs) are primary saturated fatty acids and are derivatives of protein fermentation by microbes in the distal colon [202]. They include isovalerate, isobutyrate and 2‐methylbutyrate, and are common in ruminant products such as milk, meat, and lanolin [203, 204]. BCFAs play a crucial role in the human gut in infancy [205]. BCFAs are the main component of membrane lipids in bacteria, particularly the *Bacillus*, *Lactobacillus*, and *Bifidobacterium* genera [206]. Unlike short‐chain fatty acids, there is a paucity of literature on the influence of BCFAs on host health [202]. However, there is evidence of their anti‐inflammatory activities demonstrated using *in vivo* and *in vitro* models. For instance, the BCFA diet alleviated necrotizing enterocolitis in neonatal rats, increased production of the anti‐inflammatory cytokine 1L‐10 (up to threefold) and increased the relative abundance of *Bacillus subtilis* and *Pseudomonas aeruginosa* [205]. Additionally, BCFAs dampened LPS‐induced IL‐8 mRNA expression and NFκB signaling in Caco‐2 human intestinal epithelial cell lines [207]. Assessment of fecal levels of isobutyrate, 2‐methylbutyrate or isovalerate in R6/1 HD mice at 12 weeks revealed no significant differences [38]. Thus, the influence of BCFAs on HD pathology as well as other NDs warrants further exploration.

### Short‐chain fatty acids

Short‐chain fatty acids (SCFAs) such as acetate, propionate, and butyrate are derived from the fermentation of dietary fiber and resistant starch by anaerobic intestinal bacteria [202]. SCFAs play a vital role in host immune health and metabolism, and their influence on, and alterations in, NDs have been extensively reviewed [208]. Shotgun‐sequencing metagenomics of fecal samples from 12 weeks of age did not show a significant difference in the abundance of butyrate‐producing bacteria *Roseburia intestinalis*, *Clostridium symbiosum*, and *Eubacterium rectale* relative to WT, neither did targeted metabolomic profiling of plasma from R6/1 HD mice at 12 weeks of age reveal significant alterations in SCFAs compared to WT littermate controls [31]. However, male R6/1 HD mice subjected to physical activity showed reduced fecal concentrations of butyrate and valerate [38]. A subsequent study provided evidence of elevated plasma acetate levels in R6/1 HD males at early and late stages of disease (14 and 20 weeks) relative to WT littermates and also reported a difference in propionate levels in female R6/1 HD mice at 20 weeks [1].

Low fecal and high plasma concentrations of SCFAs have been shown in PD patients and associated with gut microbiota composition and disease severity (specifically motor and cognitive function as assessed by the Movement Disorder Society–Unified Parkinson's Disease Rating Scale and MMSE respectively) [209]. Elevated levels of SCFAs have been linked to exacerbated AD pathology. Specifically, SPF APPPS1 mice showed elevated levels of acetate, propionate, and butyrate compared to GF APPPS1 mice, and supplementation with the three SCFAs significantly increased amyloid plaques in both GF and SPF mice [210]. Altered serum levels of SCFAs, notably valeric and caproic acid, have been linked to pathogenesis and cognitive impairment in schizophrenia [211].

Furthermore, decreased fecal levels of propionic, butyric, and isobutyric acid have been reported in patients with essential tremor (ET) compared to healthy subjects but lower levels of isovaleric and isobutyric acid compared to PD patients and this was associated with a decreased abundance of *Faecalibaacterium* and *Catenibacterium* in ET subjects [212]. Interestingly, a negative association between propionic acid and constipation and autonomic dysfunction was uncovered while isobutyric and isovaleric acids were negatively linked to tremor severity [212].

### Polyamines

Polyamines (PAs) such as putrescine, spermine, and spermidine are polycationic aliphatic amines derived from l‐ornithine from the decarboxylation of amino acids and play vital roles in biochemical and physiological processes [213, 214]. Polyamines can be endogenously produced, exogenously supplemented through diet, or synthesized by intestinal bacteria. Microbial polyamines regulate mucosal homeostasis, intestinal epithelial cell proliferation and barrier integrity [215, 216]. Evidence of the neuroprotective role of PAs has been provided in various NDs [217]. In the quinolinic acid (QA)‐induced excitotoxic rat model, spermidine ameliorated memory impairment in a dose‐dependent manner as assessed by the object recognition test, as well as decreased QA‐induced astrogliosis [218]. Similarly, pretreatment with spermidine decreased motor impairments, oxidative stress, and neuroinflammation in the 3‐NP model of striatal damage, in a dose‐dependent manner [219] and alleviated PD pathology in *Drosophila* and *Caenorhabditis elegans* by preventing the loss of locomotor activity (in flies) dopaminergic neurons and by inducing autophagy [220]. Conversely, alterations in PA metabolism have been implicated in the pathology of HD. Evidence of decreased spermine was found in the putamen of human HD brains and suggested to be indicative of atrophy [213]. Using *in vitro* models of HD, treatment with spermine, and spermidine but not putrescine, was found to increase mHTT aggregates [221]. Overall, elevated levels of PAs have been linked to cognitive deficits and synaptic loss in NDs including HD, AD, and PD, therefore the dual role (beneficial/deleterious) of these metabolites calls for a better understanding [214, 217, 222, 223, 224].

## Interventions targeting the gut microbiome in HD

### Psychobiotics

Probiotics are live microorganisms (e.g., bacteria, fungi) with activities beneficial to host health including suppressing the growth of pathogenic bacteria and protecting the intestinal epithelial barrier. Probiotics can have fermentative action and digest dietary fiber to release SCFAs, neurotransmitters and other biological molecules [225]. Some SCFA‐producing bacteria include *Lactobacillus*, *Bifidobacterium*, *Akkermansia*, *F. prausnitzii*, *C. leptum*, and *E. rectale*. Prebiotics on the other hand are non‐digestible high dietary fiber substances that serve as substrates for intestinal microbes which eventually convert them to SCFAs [226]. There are a plethora of studies providing consistent evidence of the efficacy of probiotics and prebiotics in modulating the gut microbiome and consequently, disease outcomes in clinical trials and preclinical models of NDs including AD, PD, and MS, and have been summarized elsewhere [227, 228]. Notably, the use of oral probiotics significantly improved constipation in PD patients as well as non‐motor symptoms of anxiety and depression [229, 230]. Moreover, foods rich in polyamines have been associated with improved cognition [231, 232], hence the increasing interest in probiotics that can elevate the abundance of polyamine‐producing gut commensals.

Wasser *et al*. [119] recently reported findings of the first randomized controlled clinical trial of a probiotic intervention in HDGECs (*n* = 41) and healthy controls (*n* = 36). The probiotic capsule was enriched with *Lactobacillus rhamnosus*, *S. cerevisiae* (*boulardii*), and *Bifidobacterium animalis* ssp *lactis* and had no adverse effects on participants. The probiotic capsule, administered for only 6 weeks, had no significant effect on gut function and dysbiosis, neither did it improve cognition and memory. However, they did observe a lower abundance of the Eggerthellaceae family in female healthy controls post‐treatment. The authors suggested the absence of a significant beneficial effect of the capsule may be attributed to the short duration of the trial among other factors. Overall, more studies are needed to identify efficacious probiotics‐based therapies for HD with minimal adverse risks.

Furthermore, there is evidence that the consumption of dietary polyphenols such as resveratrol, and curcumin can attenuate neurodegenerative pathologies, and these compounds are also classified as prebiotics [233]. They are metabolized by gut bacteria and elicit antioxidant and anti‐inflammatory actions [234]. Luteolin, a flavone richly found in fruits, vegetables, and herbs has promising benefits. Luteolin‐fed HD fruit flies showed ameliorated motor defects, reduced protein HTT aggregates, and increased survival [235]. In a dose‐dependent manner, luteolin improved spatial learning deficits (as assessed by the Morris water maze) and decreased cortical Aβ plaques in 3×Tg‐AD mice [236]. It also inhibited ER stress response and consequently neuroinflammation. Luteolin supplementation has been shown to modulate the gut microbiota. One study reported increased *Erysipelatoclostridium* and *Pseudomonas* as well as decreased *Faecalibaculum* [237] and another, increased abundance of *Lactobacillus*, *Bacteroides*, *Roseburia* and *Butyricicoccus* [238]. Interestingly, the carotenoid, crocin had a more efficacious effect compared to Luteolin in the HD flies described above [235]. Overall, the therapeutic potential of other phytoconstituents for HD, as well as the associated clinical trials, have been extensively covered [239].

### Dietary interventions

Diet‐based approaches have been considered a therapeutic strategy for HD. Here we present some of the recent evidence in support of dietary interventions.

Gubert *et al*. [240] recently discovered the promising potential of a high‐fiber (HF) diet in improving disease phenotype in the male R6/1 HD mice. HF diet improved cognition and the depressive‐like phenotype, as characterized by the novel object recognition and Porsolt swim tests respectively [240]. Notably, HF significantly improved GI health measures as evidenced by increased fecal water content, softened stool consistency, and decreased gut transit time in WT mice comparably to HD mice, suggesting ease of constipation. Additionally, HF increased colon length and caecum weight in both genotypes but only increased caecum length in WT [240].

Importantly, dietary fiber did not modulate body weight, water intake, brain weight or motor function as characterized by rotarod performance, DigiGait and limb‐clasping tests [240]. There was no significant difference in α‐diversity (richness and Shannon indices) but there was a significant difference in β‐diversity (Aitchison distance). Permutational multivariate analysis of variance (PERMANOVA) testing revealed significant interactions between genotype and diet on microbiome composition at weeks 14 and 20 [240].

Interestingly, microbiota composition between HF‐fed WT and HD mice significantly differed at 14 weeks of age. R6/1 HD mice on a HF diet mice showed an increased relative abundance of the Phyla Desulfobacterota and the families Bacteroidaceae, Butyricicoccaceae, Oscillospiraceae, Ruminococcaceae and a decreased relative abundance of the phyla Actinobacteriota, Campylobacterota, Fusobacteriota and Proteobacteria, the families Campylobacteraceae, Carnobacteriaceae, Corynebacteraceae, Gemellaceae, Micrococcaceae, Neisseriaceae, Selenomonaceae, Weeksellaceae and the order Lactobacillales [240]. Furthermore, Phylogenetic Investigation of Communities by Reconstruction of Unobserved States (PICRUST) analysis revealed a decreased expression of pathways associated with nitrate reduction and protein N‐glycosylation in HF‐fed R6/1 HD mice bacterial communities [240]. The possible pathogenic roles of dysregulations of these pathways in HD and other NDs have been extensively discussed elsewhere [241, 242, 243, 244].

A ketogenic diet (KD) involves consuming whole foods that have high fat content and low carbohydrates. KD‐induced microbiota alterations are well documented in neurological disorders including epilepsy [245], AD, and PD, but not so much in HD, and have been summarized elsewhere [246, 247]. A case study of a 41‐year‐old subject with progressive HD (47 CAG repeats) on a Time‐Restricted Ketogenic Diet (TRKD) for 48 weeks, during which the subject consumed two meals daily without snacks, provides evidence of improved motor function, quality of life and behavior problems, especially apathy. However, there was no improvement in cognition and no significant changes in weight [248]. Of course, such case studies represent low‐level evidence, and need to be followed up with well‐powered randomized controlled trials. In R6/2 HD mice, KD delayed the onset of weight loss from 9 to 11 weeks and had no adverse effect on memory, motor coordination and locomotion [249]. Interestingly, KD‐fed WT males but not females showed improved motor and locomotor function compared to non‐KD WT mice. In the BACHD model of HD, 3 months of dietary ketosis altered the microbiome with a significantly increased abundance of *Akkermansia municiphila* compared to mice on a normal diet and improved circadian and motor function [250]. It is pertinent to highlight studies with contradictory findings. For instance, Olson *et al*. [251] showed that KD exacerbated cognitive impairments in conventional SPF in a gut‐microbiota‐dependent manner, Lauritzen *et al*. reported that KD aggravated neurodegeneration in transgenic mice with hippocampal neuronal mitochondria dysfunction and Zhao *et al*. demonstrated visual–spatial memory impairment and diminished brain growth as long‐term effects of KD in a rat model of epilepsy [252, 253].

Mediterranean diet (MeDi), a centuries‐old dietary pattern, is chiefly composed of plant sources, fish, and extra virgin olive oil, with little to no inclusion of red meat, poultry, dairy products. Low adherence to the MeDi diet was associated with higher BMI and increased intake of high‐caloric meals was highlighted as a risk factor for shortening time to clinical onset of HD (phenoconversion) in premanifest gene carriers, while consuming meals with dairy products was linked with a two‐fold risk of phenoconversion [254]. In a Spanish cohort of HD and premanifest HD gene carriers, moderate adherence to MeDi was associated with positive outcomes including better quality of life, lower motor impairment and decreased risk for abdominal obesity [255]. The outcomes of other studies that investigated the effect of the MeDi diet in HD have been reviewed elsewhere [256]. Furthermore, it is still unclear whether the gut microbiota modulated this neuroprotective effect of Medi in HD, as no microbiome measures were reported. However, other studies in adult populations have reported that MeDI increased bifidobacterial counts, total SCFA [257] as well as the relative abundance of butyrate producers, *F. prausnitzii*, *Eubacterium*, *Roseburia* [258], and decreased abundance of the pathogenic colonizer *Escherichia coli* associated with inflammatory activities [259].

The neuroprotective role of intermittent fasting (IF) or time‐restricted feeding in NDs including HD in humans and experimental models has been addressed previously [260] but with no focus on alterations in microbial profiles. IF induced autophagy and promoted mHTT clearance in YAC128 HD mice [261]. Heterogenous evidence from human and animal studies suggests that IF can alter microbial diversity and composition and the relative abundance of species such as *Akkermansia*, *Faecalibacterium*, *Lactobacillus*, and *Roseburia* and can dampen the production of proinflammatory cytokines IL‐6, IL‐1β, TNF‐α [262] and even CRP [263].

### Environmental interventions and physical activity

Environmental enrichment (EE) encompasses the introduction of novel stimuli to engage sensory, cognitive, motor, and social activities, and its neuroprotective role has been extensively reviewed in previous literature. Various studies have shown the therapeutic benefits of EE and physical exercise (EX) in delaying the onset and progression of disease in R6/1 mice [264, 265]. Intriguingly, the microbiota profiles in R6/1 HD mice exposed to different housing conditions for a duration of 6 weeks, namely EE and EX, compared to standard housing (SH), exhibit distinct patterns [38]. The most notable distinguishing signatures were the bacterial orders Bacteroidales, Lachnospirales, and Oscillospirales. Briefly, in male WT mice, SH showed Coriobacteriales and Monoglobales, EE affected Oscillospirales and Lactobacillales, and EX affected Desulfovibrionales and Bacteroidales, respectively. Whereas male HD mice in SH conditions showed Coriobacteriales and Bacteroidales, EE affected Lachnospirales and Bacteroidales, and EX affected Gastranaerophilales and Oscillospirales. For female WT mice across all three housing conditions, a consistent presence of Bacteroidales and Lachnospirales was reported. In contrast, female HD mice exhibited microbiota compositions depending on the housing conditions. SH housing showed Bacteroidales and Lachnospirales, EE affected Deferribacterales and Peptostreptococcales‐Tissierellales, and EX affected Lachnospirales and Bacteroidales. Overall, the effects of lifestyle, diet, physical activity and other environmental factors on HD pathology in humans and preclinical models have been extensively reviewed elsewhere [266] and the influence of these factors on gut microbiota in NDs have been discussed [34]. However, our understanding of interactions between physical activity and HD‐specific microbiota in humans is limited, and warrants further studies.

### Antibiotics

Broad‐spectrum antibiotics (ABX) can have long‐term effects on microbial diversity, structure, and function as well as colonization periods [267, 268]. Moreover, higher oral ABX exposure has been positively associated with increased AD [269], PD [267] and ALS risk in individuals [270]. Yet, there is evidence of the neuroprotective and anti‐inflammatory activities of select antibiotics in clinical and experimental models. In transgenic fruit flies expressing the human mHTT, administration of rifaximin (broad‐spectrum antibiotic, poorly absorbed in the GIT) or 1% penicillin–streptomycin (absorbable) not only depleted the commensal bacteria but improved disease phenotype characterized by lower aggregates of amyloidogenic N‐terminal fragments of HTT compared to controls as well as delayed onset of motor deficits [271]. Interestingly, flies colonized by *Eschericia coli* showed an elevated abundance of HTT aggregates, motor defects as well and shorter lifespans [235]. In a different study, HD flies treated with rapamycin, lithium, or a combination of both were protected against neurodegeneration compared to vehicle‐treated, with the combinatorial treatment offering even more neuroprotection [272].

Minocycline, a second‐generation derivative of tetracycline, is clinically safe and crosses the blood–brain barrier. Its protective role in neurologic diseases has been demonstrated [273]. Phase one and two trials of Minocycline in HD patients (NCT00029874) are documented but the findings are unreported. Intraperitoneal administration of minocycline delayed disease progression and mortality in R6/2 HD mice [274]. It also downregulated caspase‐1 and ‐3 mRNA expression as well as inducible nitric oxide synthetase (iNOS) activation, these trio have been associated with exacerbated HD phenotype [274]. However, minocycline did not prevent weight loss or decrease fasting blood glucose levels. Similar findings in R6/2 HD mice have been demonstrated [275, 276]. Surprisingly, the combination of minocycline and dietary supplementation of coenzymeQ_10_ significantly improved body weight from 13 to 16 weeks of age [276].

The antibiotic mithramycin (MTM), now called plicamycin, is an FDA‐approved anti‐tumoral, anti‐hypercalcemia drug, naturally derived from *Streptomyces agrillaceus*. In addition to its vital role in regulating cell proliferation and differentiation, it suppresses the expression of protooncogenes including c‐myc [277]. Intraperitoneally‐MTM‐treated R6/2 HD mice showed improved survival, motor performance (rotarod) and significant resistance to brain neuropathology [278].

R6/1 HD mice administered a non‐absorbable ABX cocktail of ertapenem sodium, vancomycin hydrochloride and neomycin sulphate did not show improved motor outcomes but ABX altered gait and locomotion (increased propel‐brake ratios) in male HD mice, increased percentage of freezing over time in females as well as decreased extinction in female HD mice [1]. Furthermore, increased body weight and decreased food intake in HD males and female mice, but increased water intake in male mice only. Additionally, ABX increased fecal water content in male HD mice but did not modulate gut permeability at 20 weeks in both sexes. Also, ABX increased fecal output in HD males and increased fecal output in WT and HD females [1]. This ability of absorbable or non‐absorbable ABX to modulate disease pathology and symptoms and remodel gut resident communities is not exclusive to HD and has been demonstrated in other NDs [279, 280, 281].

The gut microbiota influence hematopoiesis (production of blood cells) by regulating type 1 interferons and consequently Signal Transducer and Activator of Transcription 1 (STAT1) signaling [282]. Thus, ABX can limit murine hematopoiesis and significantly suppress multipotent progenitors [283]. Moreover, the depletion of intestinal microbiota disrupts basal STAT1 signaling and T cell homeostasis [283]. Interestingly, these hematopoietic defects induced by ABX can be rescued by administering the microbiota‐derived product Nucleotide‐binding Oligomerization Domain‐containing protein 1 (NOD1) ligand [282].

The spleen, the largest secondary lymphoid organ, and a reservoir for immune cells such as macrophages and monocytes, plays a crucial role in hematopoiesis and regulating peripheral innate immune response [284]. These functions are heavily influenced by the gut microbiota and their secretory products as described above. In fact, the gut microbiota are significantly altered in splenectomized patients and mice [285, 286]. The most common cause of death in HD patients is pneumonia [287]. The bacterial load in the spleen has been correlated with the occurrence of bacteremia during pneumonia in humans, baboons and mice [288]. There is consistent evidence of increased splenic macrophages and lymphocytes in murine models of NDs such as AD, PD, and ALS, giving rise to a systemic proinflammatory response [289]. The genes *TNF‐α*, and *IL‐6* were upregulated in the spleens of rats with depression‐like phenotype [290].

Our understanding of the influence of ABX on the gut‐brain spleen axis in HD is limited, but studies in healthy mice have reported interesting observations. For instance, 14 days administration of a broad‐spectrum ABX cocktail consisting of ampicillin, neomycin, and metronidazole to healthy male mice decreased spleen weight and disrupted the population of splenic immune cells [291]. It also significantly decreased the expression of the microglial marker Iba1 in the cerebral cortex [291]. Untargeted metabolomics analysis of the plasma, spleen and cerebral cortex of these mice revealed altered metabolite signatures [291].

Peripheral Ly6C^high^ monocytes are versatile innate immune cells implicated in the pathogenesis of neurologic, inflammatory and cardiovascular disorders including MS, spinal cord injury, ALS, AD, PD, depression, colitis, heart ischemia and atherosclerosis [292, 293, 294, 295, 296, 297, 298, 299, 300, 301]. Pattern recognition receptors (PRR) ligands are constitutively expressed by the gut microbiota and regulate the splenic Ly6C^high^ monocytes' homeostasis and function during steady state [292]. Interestingly, 2–6 days administration of a broad‐spectrum ABX cocktail consisting of vancomycin, neomycin, and metronidazole to female mice significantly reduced the expression of PRR ligands in the serum and this decrease was associated with reduced numbers of splenic Ly6C^high^ monocytes and their disrupted activities [292]. However, this treatment did not impair hematopoiesis in the bone marrow [282]. Notably, intraperitoneal injection of PRR ligands significantly restored the population and function of splenic Ly6C^high^ monocytes in the ABX‐treated mice [292].

Overall, the microbiota–gut–brain axis in HD is a developing field of research and the sustainability and long‐term efficacy of ABX manipulation (and other manipulations) of gut microbiota in HDGECs needs to be better understood. After all, we “contain multitudes” for a reason, and any manipulation of microbiota has the potential to produce both positive and negative consequences.

### Fecal microbiota transplantation

As discussed earlier, gut dysbiosis is a causative factor in the onset and progression of neuropathological conditions. Fecal microbiota transplantation (FMT), a therapeutic strategy, involves the artificial transfer of gut microbiota from healthy subjects to disease subjects to alleviate gut dysbiosis in the recipient. Remodeling the gut microbial communities to treat diseases has been used for centuries. Hence, FMT is not a novel approach and is currently a standardized, less invasive treatment for *Clostridium difficile* infection compared to colectomy, with a Fischer study reporting an 87% cure rate [302] and a Spartz study providing more evidence [303].

Guzzardi *et al*. recently showed that cognitive phenotype is ‘transferrable’ through FMT. GF mice recipients of fecal matter from children with high cognitive performance showed superior cognitive phenotype as assessed by the Y‐maze test, while recipients of children with low cognitive scores performed poorly [186]. Similarly, FMT from young donor mice (3–4 months) not only remodeled the hippocampal metabolome and transcriptome but reversed aging‐associated markers in peripheral and hippocampal immune cells of aged recipient mice (19–20 months) and alleviated aging‐induced behavioral anomalies [33].

Interestingly, the U.S. Food and Drug Administration recently approved two drugs chiefly derived from fecal samples of healthy subjects for the treatment of *C. difficile*‐induced colitis [304, 305]. The current application of FMT in various neurological and psychiatric diseases, including AD, PD, ALS, and MS, has been extensively reviewed [306, 307, 308]. However, there is a dearth of literature on the exploration of FMT as a therapeutic tool in HD patients and experimental models.

Attempts to modulate HD disease outcome in R6/1 HD mice via FMT following antibiotics (ABX) depletion of the host gut microbiota have been made [1]. Notably, FMT from WT mice into HD mice modulated the cognitive phenotype in a sex‐specific manner, with females showing better improvement as characterized by Y‐maze and fear‐conditioning tests. FMT decreased fecal output and water content in both males and females, as well as gut transit time. Furthermore, age‐dependent, genotype, and sex‐specific changes in gut macroscopic structure with FMT treatment were demonstrated. Additionally, FMT increased fecal bacterial load at week 12 in both genotypes compared to vehicle‐treated groups. This study also demonstrated FMT's efficiency in restoring microbial richness and composition, reported poor engraftment in HD males, and suggested that this resistance to colonization could be attributed to aggravated gut pathology [1].

## Concluding remarks and future directions

Modulating the gut microbiome in HD is a promising treatment strategy. There is a paucity of literature, which underscores the need for additional studies to delineate the complexities of the microbiota–gut–brain axis in HD. Microbial profiling is crucial to elucidate gut ecological changes arising from HD interventions such as diet and physical activity. Moreover, microbiome‐drug interactions in HD require more characterization and special consideration should be given to inter‐individual gut microbiota variations as this knowledge could inform personalized medicine. Future experiments to assess microbial strain‐specific effects in HD are warranted. Additionally, there is a compelling need for a more thorough assessment of the gut mycobiome, virome, and phageome, and their metabolites, in HDGECs compared to healthy individuals, and to ultimately improve our understanding of the complex web of trans‐kingdom interactions, and their impact on HD onset and progression.

Furthermore, the link between oral dysbiosis and HD warrants delineation. As HD progresses, individuals have limited ability to maintain proper oral hygiene, thus they are more vulnerable to dental caries and periodontal diseases which can aggravate systemic inflammation and other HD symptoms. Therefore, interventions aimed at ameliorating possible oral dysbiosis in HDGECs may mediate dramatic improvement in quality of life. Importantly, characterization of the oral microbiome in these subjects could unravel microbial communities as a novel biomarker for HD.

Targeting the vagus nerve through VNS (which has anti‐inflammatory potentials) could also be of interest to restore microbiota‐gut‐brain‐axis homeostasis in disease conditions characterized by dysbiosis, intestinal inflammation and psychiatric manifestations, such as HD. Such candidate interventions need to be systematically tested in valid preclinical models.

Finally, enviromimetics [309] and their subclass, exercise mimetics [310] could be a complementary therapy for HD treatment and represent a non‐pharmacological approach to improve the quality of life of HD subjects, but further research is required to fine‐tune and personalize such interventions.

## Conflict of interest

The authors declare no conflict of interest.

## Author contributions

MNE conceptualized and wrote the first draft of the manuscript; CG and AJH provided critical feedback, edited and revised subsequent drafts. CG made the figures and graphical abstract.

## References

[1] Gubert C , Choo JM , Love CJ , Kodikara S , Masson BA , Liew JJM , Wang Y , Kong G , Narayana VK , Renoir T *et al*. (2022) Faecal microbiota transplant ameliorates gut dysbiosis and cognitive deficits in Huntington's disease mice. Brain Commun 4, fcac205. 36035436 10.1093/braincomms/fcac205 PMC9400176

[2] MacDonald ME , Ambrose CM , Duyao MP , Myers RH , Lin C , Srinidhi L , Barnes G , Taylor SA , James M , Groot N *et al*. (1993) A novel gene containing a trinucleotide repeat that is expanded and unstable on Huntington's disease chromosomes. The Huntington's Disease Collaborative Research Group. Cell 72, 971–983. 8458085 10.1016/0092-8674(93)90585-e

[3] McColgan P & Tabrizi SJ (2018) Huntington's disease: a clinical review. Eur J Neurol 25, 24–34. 28817209 10.1111/ene.13413

[4] Sharma G , Biswas SS , Mishra J , Navik U , Kandimalla R , Reddy PH , Bhatti GK & Bhatti JS (2023) Gut microbiota dysbiosis and Huntington's disease: exploring the gut‐brain axis and novel microbiota‐based interventions. Life Sci 328, 121882. 37356750 10.1016/j.lfs.2023.121882

[5] Medina A , Mahjoub Y , Shaver L & Pringsheim T (2022) Prevalence and incidence of Huntington's disease: an updated systematic review and meta‐analysis. Mov Disord 37, 2327–2335. 36161673 10.1002/mds.29228 PMC10086981

[6] Furby H , Moore S , Nordstroem AL , Houghton R , Lambrelli D , Graham S , Svenningsson P & Petersen A (2022) Comorbidities and clinical outcomes in adult‐ and juvenile‐onset Huntington's disease: a study of linked Swedish National Registries (2002‐2019). J Neurol 270, 864–876. 36253622 10.1007/s00415-022-11418-y PMC9886595

[7] Bates GP , Dorsey R , Gusella JF , Hayden MR , Kay C , Leavitt BR , Nance M , Ross CA , Scahill RI , Wetzel R *et al*. (2015) Huntington disease. Nat Rev Dis Primers 1, 15005. 27188817 10.1038/nrdp.2015.5

[8] Ross CA & Tabrizi SJ (2011) Huntington's disease: from molecular pathogenesis to clinical treatment. Lancet Neurol 10, 83–98. 21163446 10.1016/S1474-4422(10)70245-3

[9] Vonsattel JP , Myers RH , Stevens TJ , Ferrante RJ , Bird ED & Richardson EP Jr (1985) Neuropathological classification of Huntington's disease. J Neuropathol Exp Neurol 44, 559–577. 2932539 10.1097/00005072-198511000-00003

[10] Zhou X , Li G , Kaplan A , Gaschler MM , Zhang X , Hou Z , Jiang M , Zott R , Cremers S , Stockwell BR *et al*. (2018) Small molecule modulator of protein disulfide isomerase attenuates mutant huntingtin toxicity and inhibits endoplasmic reticulum stress in a mouse model of Huntington's disease. Hum Mol Genet 27, 1545–1555. 29462355 10.1093/hmg/ddy061 PMC5905666

[11] Ross CA & Poirier MA (2004) Protein aggregation and neurodegenerative disease. Nat Med 10 (Suppl), S10–S17. 15272267 10.1038/nm1066

[12] Du G , Dong W , Yang Q , Yu X , Ma J , Gu W & Huang Y (2020) Altered gut microbiota related to inflammatory responses in patients with Huntington's disease. Front Immunol 11, 603594. 33679692 10.3389/fimmu.2020.603594 PMC7933529

[13] van der Burg JM , Winqvist A , Aziz NA , Maat‐Schieman ML , Roos RA , Bates GP , Brundin P , Bjorkqvist M & Wierup N (2011) Gastrointestinal dysfunction contributes to weight loss in Huntington's disease mice. Neurobiol Dis 44, 1–8. 21624468 10.1016/j.nbd.2011.05.006

[14] Andrich JE , Wobben M , Klotz P , Goetze O & Saft C (2009) Upper gastrointestinal findings in Huntington's disease: patients suffer but do not complain. J Neural Transm (Vienna) 116, 1607–1611. 19771391 10.1007/s00702-009-0310-1

[15] Kobal J , Matej K , Koželj M & Podnar S (2018) Anorectal dysfunction in presymptomatic mutation carriers and patients with Huntington's disease. J Huntingtons Dis 7, 259–267. 29889076 10.3233/JHD-170280

[16] Trejo A , Tarrats RM , Alonso ME , Boll M‐C , Ochoa A & Velásquez L (2004) Assessment of the nutrition status of patients with Huntington's disease. Nutrition 20, 192–196. 14962685 10.1016/j.nut.2003.10.007

[17] Mesquita J , Silva L & Machado A (2010) Delayed Huntington's disease diagnosis in two alcoholic patients with a family history of “Parkinson's disease”. J Neuropsychiatry Clin Neurosci 22, 451‐a.e2–451.e2. 10.1176/jnp.2010.22.4.451.e2 21037133

[18] Mochel F , Charles P , Seguin F , Barritault J , Coussieu C , Perin L , Le Bouc Y , Gervais C , Carcelain G , Vassault A *et al*. (2007) Early energy deficit in Huntington disease: identification of a plasma biomarker traceable during disease progression. PLoS One 2, e647. 17653274 10.1371/journal.pone.0000647 PMC1919424

[19] Ramos ARS & Garrett C (2017) Huntington's disease: premotor phase. Neurodegener Dis 17, 313–322. 29073635 10.1159/000481172

[20] Aldaz T , Nigro P , Sanchez‐Gomez A , Painous C , Planellas L , Santacruz P , Camara A , Compta Y , Valldeoriola F , Marti MJ *et al*. (2019) Non‐motor symptoms in Huntington's disease: a comparative study with Parkinson's disease. J Neurol 266, 1340–1350. 30834978 10.1007/s00415-019-09263-7

[21] Cankar K , Melik Z , Kobal J & Starc V (2018) Evidence of cardiac electrical remodeling in patients with Huntington disease. Brain Behav 8, e01077. 30028085 10.1002/brb3.1077 PMC6085913

[22] Aziz NA , Anguelova GV , Marinus J , van Dijk JG & Roos RA (2010) Autonomic symptoms in patients and pre‐manifest mutation carriers of Huntington's disease. Eur J Neurol 17, 1068–1074. 20192977 10.1111/j.1468-1331.2010.02973.x

[23] Szymuś K , Bystrzyński A , Kwaśniak‐Butowska M , Konkel A , Leśnicka A , Nowacka M & Sławek J (2020) Sexual dysfunction in Huntington's disease – a systematic review. J Neurol Neurosurg 54, 305–311. 10.5603/JNNS.a2020.0025 32242915

[24] Bull MJ & Plummer NT (2014) Part 1: The human gut microbiome in health and disease. Integr Med (Encinitas) 13, 17–22. 26770121 PMC4566439

[25] Deo PN & Deshmukh R (2019) Oral microbiome: unveiling the fundamentals. J Oral Maxillofac Pathol 23, 122–128. 10.4103/jomfp.JOMFP_304_18 PMC6503789 31110428

[26] Escapa IF , Chen T , Huang Y , Gajare P , Dewhirst FE & Lemon KP (2018) New insights into human nostril microbiome from the expanded human oral microbiome database (eHOMD): a resource for the microbiome of the human aerodigestive tract. mSystems 3, e00187‐18. 10.1128/mSystems.00187-18 PMC6280432 30534599

[27] Sharma N , Bhatia S , Sodhi AS & Batra N (2018) Oral microbiome and health. AIMS Microbiol 4, 42–66. 31294203 10.3934/microbiol.2018.1.42 PMC6605021

[28] Konkel JE , O'Boyle C & Krishnan S (2019) Distal consequences of oral inflammation. Front Immunol 10, 1403. 31293577 10.3389/fimmu.2019.01403 PMC6603141

[29] Hashimoto K (2023) Emerging role of the host microbiome in neuropsychiatric disorders: overview and future directions. Mol Psychiatry 28, 3625–3637. 37845499 10.1038/s41380-023-02287-6 PMC10730413

[30] Li S , Zhao L , Xiao J , Guo Y , Fu R , Zhang Y & Xu S (2023) The gut microbiome: an important role in neurodegenerative diseases and their therapeutic advances. Mol Cell Biochem. doi: 10.1007/s11010-023-04853-6 37787835

[31] Kong G , Ellul S , Narayana VK , Kanojia K , Ha HTT , Li S , Renoir T , Cao KL & Hannan AJ (2020) An integrated metagenomics and metabolomics approach implicates the microbiota‐gut‐brain axis in the pathogenesis of Huntington's disease. Neurobiol Dis 148, 105199. 33249136 10.1016/j.nbd.2020.105199

[32] DeGruttola AK , Low D , Mizoguchi A & Mizoguchi E (2016) Current understanding of dysbiosis in disease in human and animal models. Inflamm Bowel Dis 22, 1137–1150. 27070911 10.1097/MIB.0000000000000750 PMC4838534

[33] Boehme M , Guzzetta KE , Bastiaanssen TFS , van de Wouw M , Moloney GM , Gual‐Grau A , Spichak S , Olavarria‐Ramirez L , Fitzgerald P , Morillas E *et al*. (2021) Microbiota from young mice counteracts selective age‐associated behavioral deficits. Nat Aging 1, 666–676. 37117767 10.1038/s43587-021-00093-9

[34] Gubert C , Kong G , Renoir T & Hannan AJ (2019) Exercise, diet and stress as modulators of gut microbiota: implications for neurodegenerative diseases. Neurobiol Dis 134, 104621. 31628992 10.1016/j.nbd.2019.104621

[35] Cryan JF , O'Riordan KJ , Cowan CSM , Sandhu KV , Bastiaanssen TFS , Boehme M , Codagnone MG , Cussotto S , Fulling C , Golubeva AV *et al*. (2019) The microbiota‐gut‐brain axis. Physiol Rev 99, 1877–2013. 31460832 10.1152/physrev.00018.2018

[36] Cryan JF , O'Riordan KJ , Sandhu K , Peterson V & Dinan TG (2020) The gut microbiome in neurological disorders. Lancet Neurol 19, 179–194. 31753762 10.1016/S1474-4422(19)30356-4

[37] O'Leary OF , Ogbonnaya ES , Felice D , Levone BR , Conroy LC , Fitzgerald P , Bravo JA , Forsythe P , Bienenstock J , Dinan TG *et al*. (2018) The vagus nerve modulates BDNF expression and neurogenesis in the hippocampus. Eur Neuropsychopharmacol 28, 307–316. 29426666 10.1016/j.euroneuro.2017.12.004

[38] Gubert C , Love CJ , Kodikara S , Mei Liew JJ , Renoir T , Le Cao KA & Hannan AJ (2021) Gene‐environment‐gut interactions in Huntington's disease mice are associated with environmental modulation of the gut microbiome. iScience 25, 103687. 35059604 10.1016/j.isci.2021.103687 PMC8760441

[39] Lee B , Moon KM & Kim CY (2018) Tight junction in the intestinal epithelium: its association with diseases and regulation by phytochemicals. J Immunol Res 2018, 2645465. 30648119 10.1155/2018/2645465 PMC6311762

[40] Chelakkot C , Ghim J & Ryu SH (2018) Mechanisms regulating intestinal barrier integrity and its pathological implications. Exp Mol Med 50, 1–9. 10.1038/s12276-018-0126-x PMC6095905 30115904

[41] Perez‐Pardo P , Kliest T , Dodiya HB , Broersen LM , Garssen J , Keshavarzian A & Kraneveld AD (2017) The gut‐brain axis in Parkinson's disease: possibilities for food‐based therapies. Eur J Pharmacol 817, 86–95. 28549787 10.1016/j.ejphar.2017.05.042

[42] Forsyth CB , Shannon KM , Kordower JH , Voigt RM , Shaikh M , Jaglin JA , Estes JD , Dodiya HB & Keshavarzian A (2011) Increased intestinal permeability correlates with sigmoid mucosa alpha‐synuclein staining and endotoxin exposure markers in early Parkinson's disease. PLoS One 6, e28032. 22145021 10.1371/journal.pone.0028032 PMC3228722

[43] Clairembault T , Leclair‐Visonneau L , Coron E , Bourreille A , Le Dily S , Vavasseur F , Heymann MF , Neunlist M & Derkinderen P (2015) Structural alterations of the intestinal epithelial barrier in Parkinson's disease. Acta Neuropathol Commun 3, 12. 25775153 10.1186/s40478-015-0196-0 PMC4353469

[44] Stan TL , Soylu‐Kucharz R , Burleigh S , Prykhodko O , Cao L , Franke N , Sjogren M , Haikal C , Hallenius F & Bjorkqvist M (2020) Increased intestinal permeability and gut dysbiosis in the R6/2 mouse model of Huntington's disease. Sci Rep 10, 18270. 33106549 10.1038/s41598-020-75229-9 PMC7589489

[45] Mangiarini L , Sathasivam K , Seller M , Cozens B , Harper A , Hetherington C , Lawton M , Trottier Y , Lehrach H , Davies SW *et al*. (1996) Exon 1 of the HD gene with an expanded CAG repeat is sufficient to cause a progressive neurological phenotype in transgenic mice. Cell 87, 493–506. 8898202 10.1016/s0092-8674(00)81369-0

[46] Etxeberria‐Rekalde E , Alzola‐Aldamizetxebarria S , Flunkert S , Hable I , Daurer M , Neddens J & Hutter‐Paier B (2021) Quantification of Huntington's disease related markers in the R6/2 mouse model. Front Mol Neurosci 13, 617229. 33505246 10.3389/fnmol.2020.617229 PMC7831778

[47] Wells JM , Brummer RJ , Derrien M , MacDonald TT , Troost F , Cani PD , Theodorou V , Dekker J , Meheust A , de Vos WM *et al*. (2016) Homeostasis of the gut barrier and potential biomarkers. Am J Physiol Gastrointest Liver Physiol 312, G171–G193. 27908847 10.1152/ajpgi.00048.2015 PMC5440615

[48] Ulluwishewa D , Anderson RC , McNabb WC , Moughan PJ , Wells JM & Roy NC (2011) Regulation of tight junction permeability by intestinal bacteria and dietary components. J Nutr 141, 769–776. 21430248 10.3945/jn.110.135657

[49] Sousa JA , Bernardes C , Bernardo‐Castro S , Lino M , Albino I , Ferreira L , Bras J , Guerreiro R , Tabuas‐Pereira M , Baldeiras I *et al*. (2023) Reconsidering the role of blood‐brain barrier in Alzheimer's disease: from delivery to target. Front Aging Neurosci 15, 1102809. 36875694 10.3389/fnagi.2023.1102809 PMC9978015

[50] Al‐Bachari S , Naish JH , Parker GJM , Emsley HCA & Parkes LM (2020) Blood‐brain barrier leakage is increased in Parkinson's disease. Front Physiol 11, 593026. 33414722 10.3389/fphys.2020.593026 PMC7784911

[51] Drouin‐Ouellet J , Sawiak SJ , Cisbani G , Lagace M , Kuan WL , Saint‐Pierre M , Dury RJ , Alata W , St‐Amour I , Mason SL *et al*. (2015) Cerebrovascular and blood‐brain barrier impairments in Huntington's disease: potential implications for its pathophysiology. Ann Neurol 78, 160–177. 25866151 10.1002/ana.24406

[52] Di Pardo A , Amico E , Scalabri F , Pepe G , Castaldo S , Elifani F , Capocci L , De Sanctis C , Comerci L , Pompeo F *et al*. (2017) Impairment of blood‐brain barrier is an early event in R6/2 mouse model of Huntington disease. Sci Rep 7, 41316. 28117381 10.1038/srep41316 PMC5259798

[53] Morton AJ , Glynn D , Leavens W , Zheng Z , Faull RLM , Skepper JN & Wight JM (2009) Paradoxical delay in the onset of disease caused by super‐long CAG repeat expansions in R6/2 mice. Neurobiol Dis 33, 331–341. 19130884 10.1016/j.nbd.2008.11.015

[54] Fleming MA 2nd , Ehsan L , Moore SR & Levin DE (2020) The enteric nervous system and its emerging role as a therapeutic target. Gastroenterol Res Pract 2020, 8024171. 32963521 10.1155/2020/8024171 PMC7495222

[55] Holzer P & Farzi A (2014) Neuropeptides and the microbiota‐gut‐brain axis. Adv Exp Med Biol 817, 195–219. 24997035 10.1007/978-1-4939-0897-4_9 PMC4359909

[56] Abot A , Cani PD & Knauf C (2018) Impact of intestinal peptides on the enteric nervous system: novel approaches to control glucose metabolism and food intake. Front Endocrinol (Lausanne) 9, 328. 29988396 10.3389/fendo.2018.00328 PMC6023997

[57] Singh A , Dawson TM & Kulkarni S (2021) Neurodegenerative disorders and gut‐brain interactions. J Clin Invest 131, e143775. 34196307 10.1172/JCI143775 PMC8245172

[58] Geng ZH , Zhu Y , Li QL , Zhao C & Zhou PH (2022) Enteric nervous system: the bridge between the gut microbiota and neurological disorders. Front Aging Neurosci 14, 810483. 35517052 10.3389/fnagi.2022.810483 PMC9063565

[59] Sciacca S , Favellato M , Madonna M , Metro D , Marano M & Squitieri F (2016) Early enteric neuron dysfunction in mouse and human Huntington disease. Parkinsonism Relat Disord 34, 73–74. 27836713 10.1016/j.parkreldis.2016.10.017

[60] Chassaing B , Kumar M , Baker MT , Singh V & Vijay‐Kumar M (2014) Mammalian gut immunity. Biomed J 37, 246–258. 25163502 10.4103/2319-4170.130922 PMC4714863

[61] Peterson LW & Artis D (2014) Intestinal epithelial cells: regulators of barrier function and immune homeostasis. Nat Rev Immunol 14, 141–153. 24566914 10.1038/nri3608

[62] Bjorkqvist M , Wild EJ , Thiele J , Silvestroni A , Andre R , Lahiri N , Raibon E , Lee RV , Benn CL , Soulet D *et al*. (2008) A novel pathogenic pathway of immune activation detectable before clinical onset in Huntington's disease. J Exp Med 205, 1869–1877. 18625748 10.1084/jem.20080178 PMC2525598

[63] Ellrichmann G , Reick C , Saft C & Linker RA (2013) The role of the immune system in Huntington's disease. Clin Dev Immunol 2013, 541259. 23956761 10.1155/2013/541259 PMC3727178

[64] Nayak A , Ansar R , Verma SK , Bonifati DM & Kishore U (2011) Huntington's disease: an immune perspective. Neurol Res Int 2011, 563784. 21876800 10.1155/2011/563784 PMC3163125

[65] Trager U , Andre R , Lahiri N , Magnusson‐Lind A , Weiss A , Grueninger S , McKinnon C , Sirinathsinghji E , Kahlon S , Pfister EL *et al*. (2014) HTT‐lowering reverses Huntington's disease immune dysfunction caused by NFkappaB pathway dysregulation. Brain 137, 819–833. 24459107 10.1093/brain/awt355 PMC3983408

[66] Trager U , Andre R , Magnusson‐Lind A , Miller JR , Connolly C , Weiss A , Grueninger S , Silajdzic E , Smith DL , Leavitt BR *et al*. (2014) Characterisation of immune cell function in fragment and full‐length Huntington's disease mouse models. Neurobiol Dis 73, 388–398. 25447230 10.1016/j.nbd.2014.10.012 PMC4262574

[67] Palpagama TH , Waldvogel HJ , Faull RLM & Kwakowsky A (2019) The role of microglia and astrocytes in Huntington's disease. Front Mol Neurosci 12, 258. 31708741 10.3389/fnmol.2019.00258 PMC6824292

[68] Ghilan M , Bostrom CA , Hryciw BN , Simpson JM , Christie BR & Gil‐Mohapel J (2014) YAC128 Huntington's disease transgenic mice show enhanced short‐term hippocampal synaptic plasticity early in the course of the disease. Brain Res 1581, 117–128. 24949563 10.1016/j.brainres.2014.06.011

[69] Fatoba O , Ohtake Y , Itokazu T & Yamashita T (2020) Immunotherapies in Huntington's disease and alpha‐synucleinopathies. Front Immunol 11, 337. 32161599 10.3389/fimmu.2020.00337 PMC7052383

[70] Khoshnan A , Ko J , Watkin EE , Paige LA , Reinhart PH & Patterson PH (2004) Activation of the IkappaB kinase complex and nuclear factor‐kappaB contributes to mutant huntingtin neurotoxicity. J Neurosci 24, 7999–8008. 15371500 10.1523/JNEUROSCI.2675-04.2004 PMC6729796

[71] Liu T , Zhang L , Joo D & Sun SC (2017) NF‐kappaB signaling in inflammation. Signal Transduct Target Ther 2, 17023. 29158945 10.1038/sigtrans.2017.23 PMC5661633

[72] Elliott CL , Allport VC , Loudon JA , Wu GD & Bennett PR (2001) Nuclear factor‐kappa B is essential for up‐regulation of interleukin‐8 expression in human amnion and cervical epithelial cells. Mol Hum Reprod 7, 787–790. 11470867 10.1093/molehr/7.8.787

[73] Giri R , Hoedt EC , Khushi S , Salim AA , Bergot AS , Schreiber V , Thomas R , McGuckin MA , Florin TH , Morrison M *et al*. (2022) Secreted NF‐kappaB suppressive microbial metabolites modulate gut inflammation. Cell Rep 39, 110646. 35417687 10.1016/j.celrep.2022.110646

[74] Soltani Khaboushan A , Moeinafshar A , Ersi MH , Teixeira AL , Majidi Zolbin M & Kajbafzadeh A‐M (2023) Circulating levels of inflammatory biomarkers in Huntington's disease: a systematic review and meta‐analysis. J Neuroimmunol 385, 578243. 37984118 10.1016/j.jneuroim.2023.578243

[75] Devos D , Lebouvier T , Lardeux B , Biraud M , Rouaud T , Pouclet H , Coron E , Bruley des Varannes S , Naveilhan P , Nguyen JM *et al*. (2012) Colonic inflammation in Parkinson's disease. Neurobiol Dis 50, 42–48. 23017648 10.1016/j.nbd.2012.09.007

[76] Houser MC , Chang J , Factor SA , Molho ES , Zabetian CP , Hill‐Burns EM , Payami H , Hertzberg VS & Tansey MG (2018) Stool immune profiles evince gastrointestinal inflammation in Parkinson's disease. Mov Disord 33, 793–804. 29572994 10.1002/mds.27326 PMC5992021

[77] Pathirana WGW , Chubb SP , Gillett MJ & Vasikaran SD (2018) Faecal calprotectin. Clin Biochem Rev 39, 77–90. 30828114 PMC6370282

[78] Schwiertz A , Spiegel J , Dillmann U , Grundmann D , Burmann J , Fassbender K , Schafer KH & Unger MM (2018) Fecal markers of intestinal inflammation and intestinal permeability are elevated in Parkinson's disease. Parkinsonism Relat Disord 50, 104–107. 29454662 10.1016/j.parkreldis.2018.02.022

[79] Dumitrescu L , Marta D , Danau A , Lefter A , Tulba D , Cozma L , Manole E , Gherghiceanu M , Ceafalan LC & Popescu BO (2021) Serum and fecal markers of intestinal inflammation and intestinal barrier permeability are elevated in Parkinson's disease. Front Neurosci 15, 689723. 34220443 10.3389/fnins.2021.689723 PMC8249847

[80] Mulak A , Budrewicz S , Panek‐Jeziorna M , Koszewicz M , Jasińska M , Marczak‐Karpina B , Słotwiński K , Podemski R & Paradowski L (2017) Fecal biomarkers of gut inflammation and intestinal barrier dysfunction in Parkinson's disease. Gastroenterology 152, S924.

[81] Silajdžić E , Rezeli M , Végvári Á , Lahiri N , Andre R , Magnusson‐Lind A , Nambron R , Kalliolia E , Marko‐Varga G , Warner TT *et al*. (2013) A critical evaluation of inflammatory markers in Huntington's disease plasma. J Huntingtons Dis 2, 125–134. 25063434 10.3233/JHD-130049

[82] Kim JS , Chen MH , Wang HE , Lu CL , Wang YP & Zhang B (2023) Inflammatory bowel disease and neurodegenerative diseases. Gut Liver 17, 495–504. 36843420 10.5009/gnl220523 PMC10352055

[83] Valadao PAC , Santos KBS , Ferreira EVTH , Macedo ECT , Teixeira AL , Guatimosim C & de Miranda AS (2020) Inflammation in Huntington's disease: a few new twists on an old tale. J Neuroimmunol 348, 577380. 32896821 10.1016/j.jneuroim.2020.577380

[84] Chang KH , Wu YR , Chen YC & Chen CM (2014) Plasma inflammatory biomarkers for Huntington's disease patients and mouse model. Brain Behav Immun 44, 121–127. 25266150 10.1016/j.bbi.2014.09.011

[85] Waxenbaum JA , Reddy V & Varacallo M (2023) Anatomy, autonomic nervous system. In StatPearls. StatPearls Publishing, Treasure Island, FL. 30969667

[86] Kenny BJ & Bordoni B (2023) Neuroanatomy, cranial nerve 10 (vagus nerve). In StatPearls. StatPearls Publishing, Treasure Island, FL. 30725856

[87] Agostoni E , Chinnock JE , Daly MDB & Murray JG (1957) Functional and histological studies of the vagus nerve and its branches to the heart, lungs and abdominal viscera in the cat. J Physiol 135, 182–205. 13398974 10.1113/jphysiol.1957.sp005703 PMC1358921

[88] Breit S , Kupferberg A , Rogler G & Hasler G (2018) Vagus nerve as modulator of the brain‐gut axis in psychiatric and inflammatory disorders. Front Psych 9, 44. 10.3389/fpsyt.2018.00044 PMC5859128 29593576

[89] Fülling C , Dinan TG & Cryan JF (2019) Gut microbe to brain signaling: what happens in vagus… Neuron 101, 998–1002. 30897366 10.1016/j.neuron.2019.02.008

[90] Galland L (2014) The gut microbiome and the brain. J Med Food 17, 1261–1272. 25402818 10.1089/jmf.2014.7000 PMC4259177

[91] Bonaz B , Bazin T & Pellissier S (2018) The vagus nerve at the interface of the microbiota‐gut‐brain axis. Front Neurosci 12, 49. 29467611 10.3389/fnins.2018.00049 PMC5808284

[92] Fang Y‐T , Lin Y‐T , Tseng W‐L , Tseng P , Hua G‐L , Chao Y‐J & Wu Y‐J (2023) Neuroimmunomodulation of vagus nerve stimulation and the therapeutic implications. Front Aging Neurosci 15, 1173987. 37484689 10.3389/fnagi.2023.1173987 PMC10358778

[93] Latorre R , Sternini C , De Giorgio R & Greenwood‐Van Meerveld B (2015) Enteroendocrine cells: a review of their role in brain‐gut communication. Neurogastroenterol Motil 28, 620–630. 26691223 10.1111/nmo.12754 PMC4842178

[94] Goehler LE , Gaykema RPA , Opitz N , Reddaway R , Badr N & Lyte M (2005) Activation in vagal afferents and central autonomic pathways: early responses to intestinal infection with *Campylobacter jejuni* . Brain Behav Immun 19, 334–344. 15944073 10.1016/j.bbi.2004.09.002

[95] Andrich J , Schmitz T , Saft C , Postert T , Kraus P , Epplen JT , Przuntek H & Agelink MW (2002) Autonomic nervous system function in Huntington's disease. J Neurol Neurosurg Psychiatry 72, 726–731. 12023413 10.1136/jnnp.72.6.726 PMC1737927

[96] Schultz JL , Harshman LA , Kamholz JA & Nopoulos PC (2021) Autonomic dysregulation as an early pathologic feature of Huntington disease. Auton Neurosci 231, 102775. 33571915 10.1016/j.autneu.2021.102775 PMC8176778

[97] Pellissier S , Dantzer C , Mondillon L , Trocme C , Gauchez AS , Ducros V , Mathieu N , Toussaint B , Fournier A , Canini F *et al*. (2014) Relationship between vagal tone, cortisol, TNF‐alpha, epinephrine and negative affects in Crohn's disease and irritable bowel syndrome. PLoS One 9, e105328. 25207649 10.1371/journal.pone.0105328 PMC4160179

[98] Pellissier S , Dantzer C , Canini F , Mathieu N & Bonaz B (2010) Psychological adjustment and autonomic disturbances in inflammatory bowel diseases and irritable bowel syndrome. Psychoneuroendocrinology 35, 653–662. 19910123 10.1016/j.psyneuen.2009.10.004

[99] Galts CPC , Bettio LEB , Jewett DC , Yang CC , Brocardo PS , Rodrigues ALS , Thacker JS & Gil‐Mohapel J (2019) Depression in neurodegenerative diseases: common mechanisms and current treatment options. Neurosci Biobehav Rev 102, 56–84. 30995512 10.1016/j.neubiorev.2019.04.002

[100] Alothman D , Marshall CR , Tyrrell E , Lewis S , Card T & Fogarty A (2022) Risk of mortality from suicide in patients with Huntington's disease is increased compared to the general population in England. J Neurol 269, 4436–4439. 35344078 10.1007/s00415-022-11085-z PMC9293836

[101] Liu Y , Zhao J & Guo W (2018) Emotional roles of mono‐aminergic neurotransmitters in major depressive disorder and anxiety disorders. Front Psychol 9, 2201. 30524332 10.3389/fpsyg.2018.02201 PMC6262356

[102] Bravo JA , Forsythe P , Chew MV , Escaravage E , Savignac HM , Dinan TG , Bienenstock J & Cryan JF (2011) Ingestion of *Lactobacillus* strain regulates emotional behavior and central GABA receptor expression in a mouse via the vagus nerve. Proc Natl Acad Sci USA 108, 16050–16055. 21876150 10.1073/pnas.1102999108 PMC3179073

[103] McVey Neufeld K‐A , Bienenstock J , Bharwani A , Champagne‐Jorgensen K , Mao Y , West C , Liu Y , Surette MG , Kunze W & Forsythe P (2019) Oral selective serotonin reuptake inhibitors activate vagus nerve dependent gut‐brain signalling. Sci Rep 9, 14290. 31582799 10.1038/s41598-019-50807-8 PMC6776512

[104] Siopi E , Galerne M , Rivagorda M , Saha S , Moigneu C , Moriceau S , Bigot M , Oury F & Lledo PM (2023) Gut microbiota changes require vagus nerve integrity to promote depressive‐like behaviors in mice. Mol Psychiatry 28, 3002–3012. 37131071 10.1038/s41380-023-02071-6 PMC10615761

[105] Konsman JP , Luheshi GN , Bluthé RM & Dantzer R (2000) The vagus nerve mediates behavioural depression, but not fever, in response to peripheral immune signals; a functional anatomical analysis. Eur J Neurosci 12, 4434–4446. 11122354 10.1046/j.0953-816x.2000.01319.x

[106] Zhang J , Ma L , Chang L , Pu Y , Qu Y & Hashimoto K (2020) A key role of the subdiaphragmatic vagus nerve in the depression‐like phenotype and abnormal composition of gut microbiota in mice after lipopolysaccharide administration. Transl Psychiatry 10, 186. 32518376 10.1038/s41398-020-00878-3 PMC7283282

[107] George MS & Aston‐Jones G (2010) Noninvasive techniques for probing neurocircuitry and treating illness: vagus nerve stimulation (VNS), transcranial magnetic stimulation (TMS) and transcranial direct current stimulation (tDCS). Neuropsychopharmacology 35, 301–316. 19693003 10.1038/npp.2009.87 PMC3055429

[108] Sahn B , Pascuma K , Kohn N , Tracey KJ & Markowitz JF (2023) Transcutaneous auricular vagus nerve stimulation attenuates inflammatory bowel disease in children: a proof‐of‐concept clinical trial. Bioelectron Med 9, 23. 37849000 10.1186/s42234-023-00124-3 PMC10583463

[109] Torrecillos F , Tan H , Brown P , Capone F , Ricciuti R , Di Lazzaro V & Marano M (2022) Non‐invasive vagus nerve stimulation modulates subthalamic beta activity in Parkinson's disease. Brain Stimul 15, 1513–1516. 10.1016/j.brs.2022.11.006 PMC7613925 36518556

[110] Aaronson ST , Sears P , Ruvuna F , Bunker M , Conway CR , Dougherty DD , Reimherr FW , Schwartz TL & Zajecka JM (2017) A 5‐year observational study of patients with treatment‐resistant depression treated with vagus nerve stimulation or treatment as usual: comparison of response, remission, and suicidality. Am J Psychiatry 174, 640–648. 28359201 10.1176/appi.ajp.2017.16010034

[111] Vargas‐Caballero M , Warming H , Walker R , Holmes C , Cruickshank G & Patel B (2022) Vagus nerve stimulation as a potential therapy in early Alzheimer's disease: a review. Front Hum Neurosci 16, 866434. 35572001 10.3389/fnhum.2022.866434 PMC9098960

[112] Magne F , Gotteland M , Gauthier L , Zazueta A , Pesoa S , Navarrete P & Balamurugan R (2020) The Firmicutes/Bacteroidetes ratio: a relevant marker of gut dysbiosis in obese patients? Nutrients 12, 1474. 32438689 10.3390/nu12051474 PMC7285218

[113] Stojanov S , Berlec A & Strukelj B (2020) The influence of probiotics on the Firmicutes/Bacteroidetes ratio in the treatment of obesity and inflammatory bowel disease. Microorganisms 8, 1715. 33139627 10.3390/microorganisms8111715 PMC7692443

[114] Mariat D , Firmesse O , Levenez F , Guimaraes V , Sokol H , Dore J , Corthier G & Furet JP (2009) The Firmicutes/Bacteroidetes ratio of the human microbiota changes with age. BMC Microbiol 9, 123. 19508720 10.1186/1471-2180-9-123 PMC2702274

[115] Meng C , Feng S , Hao Z , Dong C & Liu H (2022) Changes in gut microbiota composition with age and correlations with gut inflammation in rats. PLoS One 17, e0265430. 35290412 10.1371/journal.pone.0265430 PMC8923432

[116] Vaiserman A , Romanenko M , Piven L , Moseiko V , Lushchak O , Kryzhanovska N , Guryanov V & Koliada A (2020) Differences in the gut Firmicutes to Bacteroidetes ratio across age groups in healthy Ukrainian population. BMC Microbiol 20, 221. 32698765 10.1186/s12866-020-01903-7 PMC7374892

[117] Seo DO & Holtzman DM (2020) Gut microbiota: from the forgotten organ to a potential key player in the pathology of Alzheimer's disease. J Gerontol A Biol Sci Med Sci 75, 1232–1241. 31738402 10.1093/gerona/glz262 PMC7302187

[118] Wasser CI , Mercieca EC , Kong G , Hannan AJ , McKeown SJ , Glikmann‐Johnston Y & Stout JC (2020) Gut dysbiosis in Huntington's disease: associations among gut microbiota, cognitive performance and clinical outcomes. Brain Commun 2, fcaa110. 33005892 10.1093/braincomms/fcaa110 PMC7519724

[119] Wasser CI , Mercieca EC , Kong G , Hannan AJ , Allford B , McKeown SJ , Stout JC & Glikmann‐Johnston Y (2023) A randomized controlled trial of probiotics targeting gut dysbiosis in Huntington's disease. J Huntingtons Dis 12, 43–55. 37005888 10.3233/JHD-220556

[120] Cox LM , Abou‐El‐Hassan H , Maghzi AH , Vincentini J & Weiner HL (2019) The sex‐specific interaction of the microbiome in neurodegenerative diseases. Brain Res 1724, 146385. 31419428 10.1016/j.brainres.2019.146385 PMC6886714

[121] Alonso R , Pisa D & Carrasco L (2019) Brain microbiota in Huntington's disease patients. Front Microbiol 10, 2622. 31798558 10.3389/fmicb.2019.02622 PMC6861841

[122] Alonso R , Pisa D & Carrasco L (2019) Searching for bacteria in neural tissue from amyotrophic lateral sclerosis. Front Neurosci 13, 171. 30863279 10.3389/fnins.2019.00171 PMC6399391

[123] Pisa D , Alonso R , Marina AI , Rabano A & Carrasco L (2018) Human and microbial proteins from corpora Amylacea of Alzheimer's disease. Sci Rep 8, 9880. 29959356 10.1038/s41598-018-28231-1 PMC6026157

[124] Pisa D , Alonso R , Rabano A & Carrasco L (2016) Corpora Amylacea of brain tissue from neurodegenerative diseases are stained with specific antifungal antibodies. Front Neurosci 10, 86. 27013948 10.3389/fnins.2016.00086 PMC4781869

[125] Pisa D , Alonso R , Rabano A , Horst MN & Carrasco L (2016) Fungal enolase, beta‐tubulin, and chitin are detected in brain tissue from Alzheimer's disease patients. Front Microbiol 7, 1772. 27872620 10.3389/fmicb.2016.01772 PMC5097921

[126] Alonso R , Pisa D , Fernandez‐Fernandez AM , Rabano A & Carrasco L (2017) Fungal infection in neural tissue of patients with amyotrophic lateral sclerosis. Neurobiol Dis 108, 249–260. 28888971 10.1016/j.nbd.2017.09.001

[127] Alonso R , Pisa D , Rabano A , Rodal I & Carrasco L (2015) Cerebrospinal fluid from Alzheimer's disease patients contains fungal proteins and DNA. J Alzheimers Dis 47, 873–876. 26401766 10.3233/JAD-150382

[128] Pisa D , Alonso R & Carrasco L (2020) Parkinson's disease: a comprehensive analysis of fungi and bacteria in brain tissue. Int J Biol Sci 16, 1135–1152. 32174790 10.7150/ijbs.42257 PMC7053320

[129] Wells C , Brennan S , Keon M & Ooi L (2021) The role of amyloid oligomers in neurodegenerative pathologies. Int J Biol Macromol 181, 582–604. 33766600 10.1016/j.ijbiomac.2021.03.113

[130] Kong G , Cao KL , Judd LM , Li S , Renoir T & Hannan AJ (2018) Microbiome profiling reveals gut dysbiosis in a transgenic mouse model of Huntington's disease. Neurobiol Dis 135, 104268. 30194046 10.1016/j.nbd.2018.09.001

[131] Halfvarson J , Brislawn CJ , Lamendella R , Vazquez‐Baeza Y , Walters WA , Bramer LM , D'Amato M , Bonfiglio F , McDonald D , Gonzalez A *et al*. (2017) Dynamics of the human gut microbiome in inflammatory bowel disease. Nat Microbiol 2, 17004. 28191884 10.1038/nmicrobiol.2017.4 PMC5319707

[132] Bastiaanssen TFS , Gururajan A , van de Wouw M , Moloney GM , Ritz NL , Long‐Smith CM , Wiley NC , Murphy AB , Lyte JM , Fouhy F *et al*. (2020) Volatility as a concept to understand the impact of stress on the microbiome. Psychoneuroendocrinology 124, 105047. 33307493 10.1016/j.psyneuen.2020.105047

[133] Li F , Gao Y , Cheng W , Su X & Yang R (2023) Gut fungal mycobiome: a significant factor of tumor occurrence and development. Cancer Lett 569, 216302. 37451425 10.1016/j.canlet.2023.216302

[134] Iliev ID , Funari VA , Taylor KD , Nguyen Q , Reyes CN , Strom SP , Brown J , Becker CA , Fleshner PR , Dubinsky M *et al*. (2012) Interactions between commensal fungi and the C‐type lectin receptor dectin‐1 influence colitis. Science 336, 1314–1317. 22674328 10.1126/science.1221789 PMC3432565

[135] Forbes JD , Bernstein CN , Tremlett H , Van Domselaar G & Knox NC (2018) A fungal world: could the gut mycobiome be involved in neurological disease? Front Microbiol 9, 3249. 30687254 10.3389/fmicb.2018.03249 PMC6333682

[136] Pisa D , Alonso R , Rabano A , Rodal I & Carrasco L (2015) Different brain regions are infected with fungi in Alzheimer's disease. Sci Rep 5, 15015. 26468932 10.1038/srep15015 PMC4606562

[137] Parker A , James SA , Purse C , Brion A , Goldson A , Telatin A , Baker D & Carding SR (2022) Absence of bacteria permits fungal gut‐to‐brain translocation and invasion in germfree mice but ageing alone does not drive pathobiont expansion in conventionally raised mice. Front Aging Neurosci 14, 828429. 35923548 10.3389/fnagi.2022.828429 PMC9339909

[138] Sørensen AB , Harholt J & Arneborg N (2023) Application of *Yarrowia lipolytica* in fermented beverages. Front Food Sci Technol 3, doi: 10.3389/frfst.2023.1190063

[139] Reyes‐García MG , García‐Tamayo F & Hernández‐Hernández F (2012) Gamma‐aminobutyric acid (GABA) increases in vitro germ‐tube formation and phospholipase B1 mRNA expression in *Candida albicans* . Mycoscience 53, 36–39.

[140] Roshchina VV (2010) Evolutionary considerations of neurotransmitters in microbial, plant, and animal cells. In Microbial Endocrinology: Interkingdom Signaling in Infectious Disease and Health ( Lyte M & Freestone PPE , eds), pp. 17–52. Springer, New York, NY.

[141] Alam A , Levanduski E , Denz P , Villavicencio HS , Bhatta M , Alhorebi L , Zhang Y , Gomez EC , Morreale B , Senchanthisai S *et al*. (2022) Fungal mycobiome drives IL‐33 secretion and type 2 immunity in pancreatic cancer. Cancer Cell 40, 153–167.e11. 35120601 10.1016/j.ccell.2022.01.003 PMC8847236

[142] Zhao S , Shang A , Guo M , Shen L , Han Y & Huang X (2022) The advances in the regulation of immune microenvironment by *Candida albicans* and macrophage cross‐talk. Front Microbiol 13, 1029966. 36466634 10.3389/fmicb.2022.1029966 PMC9717684

[143] Zhang F , Aschenbrenner D , Yoo JY & Zuo T (2022) The gut mycobiome in health, disease, and clinical applications in association with the gut bacterial microbiome assembly. Lancet Microbe 3, e969–e983. 36182668 10.1016/S2666-5247(22)00203-8

[144] Kong G , Lê Cao K‐A & Hannan AJ (2022) Alterations in the gut fungal community in a mouse model of Huntington's disease. Microbiol Spectr 10, e0219221. 35262396 10.1128/spectrum.02192-21 PMC9045163

[145] D'Argenio V , Veneruso I , Gong C , Cecarini V , Bonfili L & Eleuteri AM (2022) Gut microbiome and mycobiome alterations in an in vivo model of Alzheimer's disease. Genes (Basel) 13, 1564. 36140732 10.3390/genes13091564 PMC9498768

[146] Vinther‐Jensen T , Bornsen L , Budtz‐Jorgensen E , Ammitzboll C , Larsen IU , Hjermind LE , Sellebjerg F & Nielsen JE (2016) Selected CSF biomarkers indicate no evidence of early neuroinflammation in Huntington disease. Neurol Neuroimmunol Neuroinflamm 3, e287. 27734023 10.1212/NXI.0000000000000287 PMC5042104

[147] Li F , Liu A , Zhao M & Luo L (2023) Astrocytic chitinase‐3‐like protein 1 in neurological diseases: potential roles and future perspectives. J Neurochem 165, 772–790. 37026513 10.1111/jnc.15824

[148] Gaur N , Huss E , Prell T , Steinbach R , Guerra J , Srivastava A , Witte OW & Grosskreutz J (2021) Monocyte‐derived macrophages contribute to chitinase dysregulation in amyotrophic lateral sclerosis: a pilot study. Front Neurol 12, 629332. 34054686 10.3389/fneur.2021.629332 PMC8160083

[149] Li C , Liang Y & Qiao Y (2022) Messengers from the gut: gut microbiota‐derived metabolites on host regulation. Front Microbiol 13, 863407. 35531300 10.3389/fmicb.2022.863407 PMC9073088

[150] Guzior DV & Quinn RA (2021) Review: microbial transformations of human bile acids. Microbiome 9, 140. 34127070 10.1186/s40168-021-01101-1 PMC8204491

[151] Ramírez‐Pérez O , Cruz‐Ramón V , Chinchilla‐López P & Méndez‐Sánchez N (2017) The role of the gut microbiota in bile acid metabolism. Ann Hepatol 16, S21–S26. 31196631 10.5604/01.3001.0010.5672

[152] Shatova OP , Zabolotneva AA & Shestopalov AV (2023) Molecular ensembles of microbiotic metabolites in carcinogenesis. Biochemistry (Mosc) 88, 867–879. 37751860 10.1134/S0006297923070027

[153] Carino A , Marchiano S , Biagioli M , Scarpelli P , Bordoni M , Di Giorgio C , Roselli R , Fiorucci C , Monti MC , Distrutti E *et al*. (2020) The bile acid activated receptors GPBAR1 and FXR exert antagonistic effects on autophagy. FASEB J 35, e21271. 10.1096/fj.202001386R 33368684

[154] Kiriyama Y & Nochi H (2023) Role of microbiota‐modified bile acids in the regulation of intracellular organelles and neurodegenerative diseases. Genes (Basel) 14, 825. 37107583 10.3390/genes14040825 PMC10137455

[155] Mulak A (2021) Bile acids as key modulators of the brain‐gut‐microbiota axis in Alzheimer's disease. J Alzheimers Dis 84, 461–477. 34569953 10.3233/JAD-210608 PMC8673511

[156] Ridlon JM , Kang DJ , Hylemon PB & Bajaj JS (2014) Bile acids and the gut microbiome. Curr Opin Gastroenterol 30, 332–338. 24625896 10.1097/MOG.0000000000000057 PMC4215539

[157] Larabi AB , Masson HLP & Baumler AJ (2023) Bile acids as modulators of gut microbiota composition and function. Gut Microbes 15, 2172671. 36740850 10.1080/19490976.2023.2172671 PMC9904317

[158] Zhang Y , Limaye PB , Renaud HJ & Klaassen CD (2014) Effect of various antibiotics on modulation of intestinal microbiota and bile acid profile in mice. Toxicol Appl Pharmacol 277, 138–145. 24657338 10.1016/j.taap.2014.03.009 PMC5533088

[159] Feng C , Zhang A , Miao J , Sun H , Han Y , Yan G , Wu F & Wang X (2018) Recent advances in understanding cross‐talk between bile acids and gut microbiota. Open J Proteomics Genomics 3, 24–34.

[160] Out C , Patankar JV , Doktorova M , Boesjes M , Bos T , de Boer S , Havinga R , Wolters H , Boverhof R , van Dijk TH *et al*. (2015) Gut microbiota inhibit Asbt‐dependent intestinal bile acid reabsorption via Gata4. J Hepatol 63, 697–704. 26022694 10.1016/j.jhep.2015.04.030 PMC5293168

[161] Mano N , Goto T , Uchida M , Nishimura K , Ando M , Kobayashi N & Goto J (2003) Presence of protein‐bound unconjugated bile acids in the cytoplasmic fraction of rat brain. J Lipid Res 45, 295–300. 14617741 10.1194/jlr.M300369-JLR200

[162] Keene CD , Rodrigues CM , Eich T , Linehan‐Stieers C , Abt A , Kren BT , Steer CJ & Low WC (2001) A bile acid protects against motor and cognitive deficits and reduces striatal degeneration in the 3‐nitropropionic acid model of Huntington's disease. Exp Neurol 171, 351–360. 11573988 10.1006/exnr.2001.7755

[163] Baloni P , Funk CC , Yan J , Yurkovich JT , Kueider‐Paisley A , Nho K , Heinken A , Jia W , Mahmoudiandehkordi S , Louie G *et al*. (2020) Metabolic network analysis reveals altered bile acid synthesis and metabolism in Alzheimer's disease. Cell Rep Med 1, 100138. 33294859 10.1016/j.xcrm.2020.100138 PMC7691449

[164] Vang S , Longley K , Steer CJ & Low WC (2014) The unexpected uses of urso‐ and tauroursodeoxycholic acid in the treatment of non‐liver diseases. Glob Adv Health Med 3, 58–69. 24891994 10.7453/gahmj.2014.017 PMC4030606

[165] Zangerolamo L , Vettorazzi JF , Rosa LRO , Carneiro EM & Barbosa HCL (2021) The bile acid TUDCA and neurodegenerative disorders: an overview. Life Sci 272, 119252. 33636170 10.1016/j.lfs.2021.119252

[166] Reddy PH (2014) Increased mitochondrial fission and neuronal dysfunction in Huntington's disease: implications for molecular inhibitors of excessive mitochondrial fission. Drug Discov Today 19, 951–955. 24681059 10.1016/j.drudis.2014.03.020 PMC4191657

[167] Rodrigues CM , Sola S , Nan Z , Castro RE , Ribeiro PS , Low WC & Steer CJ (2003) Tauroursodeoxycholic acid reduces apoptosis and protects against neurological injury after acute hemorrhagic stroke in rats. Proc Natl Acad Sci USA 100, 6087–6092. 12721362 10.1073/pnas.1031632100 PMC156330

[168] Keene CD , Rodrigues CM , Eich T , Chhabra MS , Steer CJ & Low WC (2002) Tauroursodeoxycholic acid, a bile acid, is neuroprotective in a transgenic animal model of Huntington's disease. Proc Natl Acad Sci USA 99, 10671–10676. 12149470 10.1073/pnas.162362299 PMC125009

[169] Ackerman HD & Gerhard GS (2016) Bile acids in neurodegenerative disorders. Front Aging Neurosci 8, 263. 27920719 10.3389/fnagi.2016.00263 PMC5118426

[170] Dionisio PA , Amaral JD , Ribeiro MF , Lo AC , D'Hooge R & Rodrigues CM (2014) Amyloid‐beta pathology is attenuated by tauroursodeoxycholic acid treatment in APP/PS1 mice after disease onset. Neurobiol Aging 36, 228–240. 25443293 10.1016/j.neurobiolaging.2014.08.034

[171] Hurley MJ , Bates R , Macnaughtan J & Schapira AHV (2022) Bile acids and neurological disease. Pharmacol Ther 240, 108311. 36400238 10.1016/j.pharmthera.2022.108311

[172] Pan X , Elliott CT , McGuinness B , Passmore P , Kehoe PG , Holscher C , McClean PL , Graham SF & Green BD (2017) Metabolomic profiling of bile acids in clinical and experimental samples of Alzheimer's disease. Metabolites 7, 28. 28629125 10.3390/metabo7020028 PMC5487999

[173] Yamauchi T , Oi A , Kosakamoto H , Akuzawa‐Tokita Y , Murakami T , Mori H , Miura M & Obata F (2020) Gut bacterial species distinctively impact host purine metabolites during aging in Drosophila. iScience 23, 101477. 32916085 10.1016/j.isci.2020.101477 PMC7520893

[174] Tong S , Zhang P , Cheng Q , Chen M , Chen X , Wang Z , Lu X & Wu H (2022) The role of gut microbiota in gout: is gut microbiota a potential target for gout treatment. Front Cell Infect Microbiol 12, 1051682. 36506033 10.3389/fcimb.2022.1051682 PMC9730829

[175] Ahn YJ , Park SJ , Woo H , Lee HE , Kim HJ , Kwon G , Gao Q , Jang DS & Ryu JH (2013) Effects of allantoin on cognitive function and hippocampal neurogenesis. Food Chem Toxicol 64, 210–216. 24296131 10.1016/j.fct.2013.11.033

[176] Lim MY , Rho M , Song YM , Lee K , Sung J & Ko G (2014) Stability of gut enterotypes in Korean monozygotic twins and their association with biomarkers and diet. Sci Rep 4, 7348. 25482875 10.1038/srep07348 PMC4258686

[177] Crane JK , Naeher TM , Broome JE & Boedeker EC (2013) Role of host xanthine oxidase in infection due to enteropathogenic and Shiga‐toxigenic *Escherichia coli* . Infect Immun 81, 1129–1139. 23340314 10.1128/IAI.01124-12 PMC3639607

[178] Handley RR , Reid SJ , Brauning R , Maclean P , Mears ER , Fourie I , Patassini S , Cooper GJS , Rudiger SR , McLaughlan CJ *et al*. (2017) Brain urea increase is an early Huntington's disease pathogenic event observed in a prodromal transgenic sheep model and HD cases. Proc Natl Acad Sci USA 114, E11293–E11302. 29229845 10.1073/pnas.1711243115 PMC5748180

[179] Toczek M , Zielonka D , Zukowska P , Marcinkowski JT , Slominska E , Isalan M , Smolenski RT & Mielcarek M (2016) An impaired metabolism of nucleotides underpins a novel mechanism of cardiac remodeling leading to Huntington's disease related cardiomyopathy. Biochim Biophys Acta 1862, 2147–2157. 27568644 10.1016/j.bbadis.2016.08.019

[180] Herman S , Niemela V , Emami Khoonsari P , Sundblom J , Burman J , Landtblom AM , Spjuth O , Nyholm D & Kultima K (2019) Alterations in the tyrosine and phenylalanine pathways revealed by biochemical profiling in cerebrospinal fluid of Huntington's disease subjects. Sci Rep 9, 4129. 30858393 10.1038/s41598-019-40186-5 PMC6411723

[181] Ajitkumar A & De Jesus O (2023) Huntington disease. In StatPearls. StatPearls Publishing, Treasure Island, FL. 32644592

[182] Illes P , Ulrich H , Chen JF & Tang Y (2023) Purinergic receptors in cognitive disturbances. Neurobiol Dis 185, 106229. 37453562 10.1016/j.nbd.2023.106229

[183] Diaz‐Hernandez M , Diez‐Zaera M , Sanchez‐Nogueiro J , Gomez‐Villafuertes R , Canals JM , Alberch J , Miras‐Portugal MT & Lucas JJ (2009) Altered P2X7‐receptor level and function in mouse models of Huntington's disease and therapeutic efficacy of antagonist administration. FASEB J 23, 1893–1906. 19171786 10.1096/fj.08-122275

[184] Olla I , Santos‐Galindo M , Elorza A & Lucas JJ (2020) P2X7 receptor upregulation in Huntington's disease brains. Front Mol Neurosci 13, 567430. 33122998 10.3389/fnmol.2020.567430 PMC7573237

[185] Li W , Silva HB , Real J , Wang YM , Rial D , Li P , Payen MP , Zhou Y , Muller CE , Tome AR *et al*. (2015) Inactivation of adenosine A2A receptors reverses working memory deficits at early stages of Huntington's disease models. Neurobiol Dis 79, 70–80. 25892655 10.1016/j.nbd.2015.03.030

[186] Angela Guzzardi M , La Rosa F , Granziera F , Panetta D , Pardo‐Tendero M , Barone M , Turroni S , Faita F , Kusmic C , Brigidi P *et al*. (2023) Gut‐derived metabolites mediating cognitive development in 5‐year‐old children: early‐life transplant in mice has lasting effects throughout adulthood. Brain Behav Immun 114, 94–110. 37557963 10.1016/j.bbi.2023.08.009

[187] Alonso‐Andres P , Albasanz JL , Ferrer I & Martin M (2018) Purine‐related metabolites and their converting enzymes are altered in frontal, parietal and temporal cortex at early stages of Alzheimer's disease pathology. Brain Pathol 28, 933–946. 29363833 10.1111/bpa.12592 PMC8028663

[188] Mielcarek M , Smolenski RT & Isalan M (2017) Transcriptional signature of an altered purine metabolism in the skeletal muscle of a Huntington's disease mouse model. Front Physiol 8, 127. 28303108 10.3389/fphys.2017.00127 PMC5332388

[189] Tomczyk M , Glaser T , Slominska EM , Ulrich H & Smolenski RT (2021) Purine nucleotides metabolism and signaling in Huntington's disease: search for a target for novel therapies. Int J Mol Sci 22, 6545. 34207177 10.3390/ijms22126545 PMC8234552

[190] Gojda J & Cahova M (2021) Gut microbiota as the link between elevated BCAA serum levels and insulin resistance. Biomolecules 11, 1414. 34680047 10.3390/biom11101414 PMC8533624

[191] Shida Y , Endo H , Owada S , Inagaki Y , Sumiyoshi H , Kamiya A , Eto T & Tatemichi M (2021) Branched‐chain amino acids govern the high learning ability phenotype in Tokai high avoider (THA) rats. Sci Rep 11, 23104. 34845278 10.1038/s41598-021-02591-7 PMC8630195

[192] Sanguinetti E , Collado MC , Marrachelli VG , Monleon D , Selma‐Royo M , Pardo‐Tendero MM , Burchielli S & Iozzo P (2018) Microbiome‐metabolome signatures in mice genetically prone to develop dementia, fed a normal or fatty diet. Sci Rep 8, 4907. 29559675 10.1038/s41598-018-23261-1 PMC5861049

[193] Toledo JB , Arnold M , Kastenmuller G , Chang R , Baillie RA , Han X , Thambisetty M , Tenenbaum JD , Suhre K , Thompson JW *et al*. (2017) Metabolic network failures in Alzheimer's disease: a biochemical road map. Alzheimers Dement 13, 965–984. 28341160 10.1016/j.jalz.2017.01.020 PMC5866045

[194] Skene DJ , Middleton B , Fraser CK , Pennings JL , Kuchel TR , Rudiger SR , Bawden CS & Morton AJ (2017) Metabolic profiling of presymptomatic Huntington's disease sheep reveals novel biomarkers. Sci Rep 7, 43030. 28223686 10.1038/srep43030 PMC5320451

[195] Cheng ML , Chang KH , Wu YR & Chen CM (2016) Metabolic disturbances in plasma as biomarkers for Huntington's disease. J Nutr Biochem 31, 38–44. 27133422 10.1016/j.jnutbio.2015.12.001

[196] Mochel F , Benaich S , Rabier D & Durr A (2011) Validation of plasma branched chain amino acids as biomarkers in Huntington disease. Arch Neurol 68, 265–267. 21320997 10.1001/archneurol.2010.358

[197] Castilhos RM , Augustin MC , Santos JAD , Pedroso JL , Barsottini O , Saba R , Ferraz HB , Vargas FR , Furtado GV , Polese‐Bonatto M *et al*. (2020) Free carnitine and branched chain amino acids are not good biomarkers in Huntington's disease. Arq Neuropsiquiatr 78, 81–87. 32159721 10.1590/0004-282X20190152

[198] Andersen JV , Skotte NH , Aldana BI , Norremolle A & Waagepetersen HS (2019) Enhanced cerebral branched‐chain amino acid metabolism in R6/2 mouse model of Huntington's disease. Cell Mol Life Sci 76, 2449–2461. 30830240 10.1007/s00018-019-03051-2 PMC11105563

[199] Polis B & Samson AO (2020) Role of the metabolism of branched‐chain amino acids in the development of Alzheimer's disease and other metabolic disorders. Neural Regen Res 15, 1460–1470. 31997805 10.4103/1673-5374.274328 PMC7059578

[200] Yoo HS , Shanmugalingam U & Smith PD (2022) Potential roles of branched‐chain amino acids in neurodegeneration. Nutrition 103–104, 111762. 10.1016/j.nut.2022.111762 35843039

[201] Zhang Y , He X , Qian Y , Xu S , Mo C , Yan Z , Yang X & Xiao Q (2022) Plasma branched‐chain and aromatic amino acids correlate with the gut microbiota and severity of Parkinson's disease. NPJ Parkinsons Dis 8, 48. 35449203 10.1038/s41531-022-00312-z PMC9023571

[202] Salazar N , González S , de los Reyes Gavilan CG & Rios‐Covian D (2022) Branched short‐chain fatty acids as biological indicators of microbiota health and links with anthropometry. In Biomarkers in Nutrition ( Patel VB & Preedy VR , eds), pp. 1–17. Springer, Cham.

[203] Ezzine C , Loison L , Montbrion N , Bôle‐Feysot C , Déchelotte P , Coëffier M & Ribet D (2022) Fatty acids produced by the gut microbiota dampen host inflammatory responses by modulating intestinal SUMOylation. Gut Microbes 14, 2108280. 35978476 10.1080/19490976.2022.2108280 PMC9466625

[204] Yao L & Hammond EG (2006) Isolation and melting properties of branched‐chain esters from lanolin. J Am Oil Chem Soc 83, 547–552.

[205] Ran‐Ressler RR , Khailova L , Arganbright KM , Adkins‐Rieck CK , Jouni ZE , Koren O , Ley RE , Brenna JT & Dvorak B (2011) Branched chain fatty acids reduce the incidence of necrotizing enterocolitis and alter gastrointestinal microbial ecology in a neonatal rat model. PLoS One 6, e29032. 22194981 10.1371/journal.pone.0029032 PMC3237582

[206] Ran‐Ressler RR , Bae S , Lawrence P , Wang DH & Brenna JT (2014) Branched‐chain fatty acid content of foods and estimated intake in the USA. Br J Nutr 112, 565–572. 24830474 10.1017/S0007114514001081 PMC4381348

[207] Yan Y , Wang Z , Greenwald J , Kothapalli KS , Park HG , Liu R , Mendralla E , Lawrence P , Wang X & Brenna JT (2016) BCFA suppresses LPS induced IL‐8 mRNA expression in human intestinal epithelial cells. Prostaglandins Leukot Essent Fatty Acids 116, 27–31. 28088291 10.1016/j.plefa.2016.12.001

[208] Guo C , Huo YJ , Li Y , Han Y & Zhou D (2022) Gut‐brain axis: focus on gut metabolites short‐chain fatty acids. World J Clin Cases 10, 1754–1763. 35317140 10.12998/wjcc.v10.i6.1754 PMC8891794

[209] Chen SJ , Chen CC , Liao HY , Lin YT , Wu YW , Liou JM , Wu MS , Kuo CH & Lin CH (2022) Association of fecal and plasma levels of short‐chain fatty acids with gut microbiota and clinical severity in patients with Parkinson disease. Neurology 98, e848–e858. 34996879 10.1212/WNL.0000000000013225 PMC8883514

[210] Colombo AV , Sadler RK , Llovera G , Singh V , Roth S , Heindl S , Sebastian Monasor L , Verhoeven A , Peters F , Parhizkar S *et al*. (2021) Microbiota‐derived short chain fatty acids modulate microglia and promote Abeta plaque deposition. Elife 10, e59826. 33845942 10.7554/eLife.59826 PMC8043748

[211] Peng H , Ouyang L , Li D , Li Z , Yuan L , Fan L , Liao A , Li J , Wei Y , Yang Z *et al*. (2022) Short‐chain fatty acids in patients with schizophrenia and ultra‐high risk population. Front Psych 13, 977538. 10.3389/fpsyt.2022.977538 PMC9790925 36578297

[212] Huang P , Zhang P , Du J , Gao C , Liu J , Tan Y & Chen S (2023) Association of fecal short‐chain fatty acids with clinical severity and gut microbiota in essential tremor and its difference from Parkinson's disease. NPJ Parkinsons Dis 9, 115. 37460569 10.1038/s41531-023-00554-5 PMC10352256

[213] Vivó M , de Vera N , Cortés R , Mengod G , Camón L & Martínez E (2001) Polyamines in the basal ganglia of human brain. Influence of aging and degenerative movement disorders. Neurosci Lett 18, 107–111. 10.1016/s0304-3940(01)01776-1 11335066

[214] Sagar NA , Tarafdar S , Agarwal S , Tarafdar A & Sharma S (2021) Polyamines: functions, metabolism, and role in human disease management. Med Sci (Basel) 9, 44. 34207607 10.3390/medsci9020044 PMC8293435

[215] Nakamura A , Kurihara S , Takahashi D , Ohashi W , Nakamura Y , Kimura S , Onuki M , Kume A , Sasazawa Y , Furusawa Y *et al*. (2021) Symbiotic polyamine metabolism regulates epithelial proliferation and macrophage differentiation in the colon. Nat Commun 12, 2105. 33833232 10.1038/s41467-021-22212-1 PMC8032791

[216] Rao JN , Xiao L & Wang JY (2020) Polyamines in gut epithelial renewal and barrier function. Physiology (Bethesda) 35, 328–337. 32783609 10.1152/physiol.00011.2020 PMC7642847

[217] Makletsova MG , Rikhireva GT , Kirichenko EY , Trinitatsky IY , Vakulenko MY & Ermakov AM (2022) The role of polyamines in the mechanisms of cognitive impairment. Neurochem J 16, 283–294.

[218] Velloso NA , Dalmolin GD , Gomes GM , Rubin MA , Canas PM , Cunha RA & Mello CF (2009) Spermine improves recognition memory deficit in a rodent model of Huntington's disease. Neurobiol Learn Mem 92, 574–580. 19632348 10.1016/j.nlm.2009.07.006

[219] Jamwal S & Kumar P (2015) Spermidine ameliorates 3‐nitropropionic acid (3‐NP)‐induced striatal toxicity: possible role of oxidative stress, neuroinflammation, and neurotransmitters. Physiol Behav 155, 180–187. 26703234 10.1016/j.physbeh.2015.12.015

[220] Buttner S , Broeskamp F , Sommer C , Markaki M , Habernig L , Alavian‐Ghavanini A , Carmona‐Gutierrez D , Eisenberg T , Michael E , Kroemer G *et al*. (2014) Spermidine protects against alpha‐synuclein neurotoxicity. Cell Cycle 13, 3903–3908. 25483063 10.4161/15384101.2014.973309 PMC4614020

[221] Tunali NE & Tüfekçi MA (2018) A26 polyamine metabolism in Huntington's disease. J Neurol Neurosurg Psychiatry 89, A9.

[222] Vrijsen S , Houdou M , Cascalho A , Eggermont J & Vangheluwe P (2023) Polyamines in Parkinson's disease: balancing between neurotoxicity and neuroprotection. Annu Rev Biochem 92, 435–464. 37018845 10.1146/annurev-biochem-071322-021330

[223] Colton CA , Xu Q , Burke JR , Bae SY , Wakefield JK , Nair A , Strittmatter WJ & Vitek MP (2004) Disrupted spermine homeostasis: a novel mechanism in polyglutamine‐mediated aggregation and cell death. J Neurosci 24, 7118–7127. 15306645 10.1523/JNEUROSCI.1233-04.2004 PMC6729181

[224] Wortha SM , Frenzel S , Bahls M , Habes M , Wittfeld K , Van der Auwera S , Bulow R , Zylla S , Friedrich N , Nauck M *et al*. (2022) Association of spermidine plasma levels with brain aging in a population‐based study. Alzheimers Dement 19, 1832–1840. 36321615 10.1002/alz.12815 PMC11246659

[225] Silva YP , Bernardi A & Frozza RL (2020) The role of short‐chain fatty acids from gut microbiota in gut‐brain communication. Front Endocrinol 11, 25. 10.3389/fendo.2020.00025 PMC7005631 32082260

[226] Portincasa P , Bonfrate L , Vacca M , De Angelis M , Farella I , Lanza E , Khalil M , Wang DQ , Sperandio M & Di Ciaula A (2022) Gut microbiota and short chain fatty acids: implications in glucose homeostasis. Int J Mol Sci 23, 1105. 35163038 10.3390/ijms23031105 PMC8835596

[227] Peterson CT (2020) Dysfunction of the microbiota‐gut‐brain axis in neurodegenerative disease: the promise of therapeutic modulation with prebiotics, medicinal herbs, probiotics, and synbiotics. J Evid Based Integr Med 25, 2515690X20957225. 10.1177/2515690X20957225 PMC7586271 33092396

[228] Aghamohammad S , Hafezi A & Rohani M (2023) Probiotics as functional foods: how probiotics can alleviate the symptoms of neurological disabilities. Biomed Pharmacother 163, 114816. 37150033 10.1016/j.biopha.2023.114816

[229] Ghalandari N , Assarzadegan F , Mahdavi H , Jamshidi E & Esmaily H (2023) Evaluating the effectiveness of probiotics in relieving constipation in Parkinson's disease: a systematic review and meta‐analysis. Heliyon 9, e14312. 36938477 10.1016/j.heliyon.2023.e14312 PMC10015253

[230] Chu C , Yu L , Li Y , Guo H , Zhai Q , Chen W & Tian F (2023) Meta‐analysis of randomized controlled trials of the effects of probiotics in Parkinson's disease. Food Funct 14, 3406–3422. 36974511 10.1039/d2fo03825k

[231] Schroeder S , Hofer SJ , Zimmermann A , Pechlaner R , Dammbrueck C , Pendl T , Marcello GM , Pogatschnigg V , Bergmann M , Müller M *et al*. (2021) Dietary spermidine improves cognitive function. Cell Rep 35, 108985. 33852843 10.1016/j.celrep.2021.108985

[232] Wirth M , Schwarz C , Benson G , Horn N , Buchert R , Lange C , Köbe T , Hetzer S , Maglione M , Michael E *et al*. (2019) Effects of spermidine supplementation on cognition and biomarkers in older adults with subjective cognitive decline (SmartAge)—study protocol for a randomized controlled trial. Alzheimers Res Ther 11, 36. 31039826 10.1186/s13195-019-0484-1 PMC6492385

[233] Islam F , Islam MM , Khan Meem AF , Nafady MH , Islam MR , Akter A , Mitra S , Alhumaydhi FA , Emran TB , Khusro A *et al*. (2022) Multifaceted role of polyphenols in the treatment and management of neurodegenerative diseases. Chemosphere 307, 136020. 35985383 10.1016/j.chemosphere.2022.136020

[234] Liu S , Cheng L , Liu Y , Zhan S , Wu Z & Zhang X (2023) Relationship between dietary polyphenols and gut microbiota: new clues to improve cognitive disorders, mood disorders and circadian rhythms. Foods 12, 1309. 36981235 10.3390/foods12061309 PMC10048542

[235] Chongtham A , Yoo JH , Chin TM , Akingbesote ND , Huda A , Marsh JL & Khoshnan A (2022) Gut bacteria regulate the pathogenesis of Huntington's disease in Drosophila model. Front Neurosci 16, 902205. 35757549 10.3389/fnins.2022.902205 PMC9215115

[236] Kou JJ , Shi JZ , He YY , Hao JJ , Zhang HY , Luo DM , Song JK , Yan Y , Xie XM , Du GH *et al*. (2021) Luteolin alleviates cognitive impairment in Alzheimer's disease mouse model via inhibiting endoplasmic reticulum stress‐dependent neuroinflammation. Acta Pharmacol Sin 43, 840–849. 34267346 10.1038/s41401-021-00702-8 PMC8975883

[237] Guo W , Luo L , Meng Y , Chen W , Yu L , Zhang C , Qiu Z & Cao P (2022) Luteolin alleviates methionine‐choline‐deficient diet‐induced non‐alcoholic steatohepatitis by modulating host serum metabolome and gut microbiome. Front Nutr 9, 936237. 35990349 10.3389/fnut.2022.936237 PMC9389599

[238] Li B , Du P , Du Y , Zhao D , Cai Y , Yang Q & Guo Z (2021) Luteolin alleviates inflammation and modulates gut microbiota in ulcerative colitis rats. Life Sci 269, 119008. 33434535 10.1016/j.lfs.2020.119008

[239] Gupta M , Sanjana , Singh N , Singh B & Alam P (2022) Role of natural products in alleviation of Huntington's disease: an overview. S Afr J Bot 151, 263–276.

[240] Gubert C , Kong G , Costello C , Adams CD , Masson BA , Qin W , Choo J , Narayana VK , Rogers G , Renoir T *et al*. (2023) Dietary fibre confers therapeutic effects in a preclinical model of Huntington's disease. Brain Behav Immun 116, 404–418. 38142919 10.1016/j.bbi.2023.12.023

[241] Deckel AW (2001) Nitric oxide and nitric oxide synthase in Huntington's disease. J Neurosci Res 64, 99–107. 11288139 10.1002/jnr.1057

[242] Kumar P , Kalonia H & Kumar A (2010) Nitric oxide mechanism in the protective effect of antidepressants against 3‐nitropropionic acid‐induced cognitive deficit, glutathione and mitochondrial alterations in animal model of Huntington's disease. Behav Pharmacol 21, 217–230. 20480544 10.1097/fbp.0b013e32833a5bf4

[243] Moll T , Shaw PJ & Cooper‐Knock J (2019) Disrupted glycosylation of lipids and proteins is a cause of neurodegeneration. Brain 143, 1332–1340. 10.1093/brain/awz358 PMC7241952 31724708

[244] Wang W , Gopal S , Pocock R & Xiao Z (2019) Glycan mimetics from natural products: new therapeutic opportunities for neurodegenerative disease. Molecules 24, 4604. 31888221 10.3390/molecules24244604 PMC6943557

[245] Shearer J , Scantlebury MH , Rho JM , Tompkins TA & Mu C (2023) Intermittent vs continuous ketogenic diet: impact on seizures, gut microbiota, and mitochondrial metabolism. Epilepsia 64, e177–e183. 37335622 10.1111/epi.17688

[246] Gough SM , Casella A , Ortega KJ & Hackam AS (2021) Neuroprotection by the ketogenic diet: evidence and controversies. Front Nutr 8, 782657. 34888340 10.3389/fnut.2021.782657 PMC8650112

[247] Kaviyarasan S , Chung Sia EL , Retinasamy T , Arulsamy A & Shaikh MF (2022) Regulation of gut microbiome by ketogenic diet in neurodegenerative diseases: a molecular crosstalk. Front Aging Neurosci 14, 1015837. 36313018 10.3389/fnagi.2022.1015837 PMC9614261

[248] Phillips MCL , McManus EJ , Brinkhuis M & Romero‐Ferrando B (2022) Time‐restricted ketogenic diet in Huntington's disease: a case study. Front Behav Neurosci 16, 931636. 35967897 10.3389/fnbeh.2022.931636 PMC9372583

[249] Ruskin DN , Ross JL , Kawamura M Jr , Ruiz TL , Geiger JD & Masino SA (2011) A ketogenic diet delays weight loss and does not impair working memory or motor function in the R6/2 1J mouse model of Huntington's disease. Physiol Behav 103, 501–507. 21501628 10.1016/j.physbeh.2011.04.001 PMC3107892

[250] Whittaker DS , Tamai TK , Bains RS , Villanueva SAM , Luk SHC , Dell'Angelica D , Block GD , Ghiani CA & Colwell CS (2022) Dietary ketosis improves circadian dysfunction as well as motor symptoms in the BACHD mouse model of Huntington's disease. Front Nutr 9, 1034743. 36407529 10.3389/fnut.2022.1034743 PMC9669764

[251] Olson CA , Iniguez AJ , Yang GE , Fang P , Pronovost GN , Jameson KG , Rendon TK , Paramo J , Barlow JT , Ismagilov RF *et al*. (2021) Alterations in the gut microbiota contribute to cognitive impairment induced by the ketogenic diet and hypoxia. Cell Host Microbe 29, 1378–1392.e6. 34358434 10.1016/j.chom.2021.07.004 PMC8429275

[252] Lauritzen KH , Hasan‐Olive MM , Regnell CE , Kleppa L , Scheibye‐Knudsen M , Gjedde A , Klungland A , Bohr VA , Storm‐Mathisen J & Bergersen LH (2016) A ketogenic diet accelerates neurodegeneration in mice with induced mitochondrial DNA toxicity in the forebrain. Neurobiol Aging 48, 34–47. 27639119 10.1016/j.neurobiolaging.2016.08.005 PMC5629920

[253] Zhao Q , Stafstrom CE , Fu DD , Hu Y & Holmes GL (2004) Detrimental effects of the ketogenic diet on cognitive function in rats. Pediatr Res 55, 498–506. 14711901 10.1203/01.PDR.0000112032.47575.D1

[254] Marder K , Gu Y , Eberly S , Tanner CM , Scarmeas N , Oakes D , Shoulson I & Huntington Study Group PHAROS Investigators (2013) Relationship of Mediterranean diet and caloric intake to phenoconversion in Huntington disease. JAMA Neurol 70, 1382–1388. 24000094 10.1001/jamaneurol.2013.3487 PMC4040231

[255] Rivadeneyra J , Cubo E , Gil C , Calvo S , Mariscal N & Martínez A (2016) Factors associated with Mediterranean diet adherence in Huntington's disease. Clin Nutr ESPEN 12, e7–e13. 28531758 10.1016/j.clnesp.2016.01.001

[256] Christodoulou CC , Demetriou CA & Zamba‐Papanicolaou E (2020) Dietary intake, Mediterranean diet adherence and caloric intake in Huntington's disease: a review. Nutrients 12, 2946. 32992896 10.3390/nu12102946 PMC7601299

[257] Garcia‐Mantrana I , Selma‐Royo M , Alcantara C & Collado MC (2018) Shifts on gut microbiota associated to Mediterranean diet adherence and specific dietary intakes on general adult population. Front Microbiol 9, 890. 29867803 10.3389/fmicb.2018.00890 PMC5949328

[258] Ghosh TS , Rampelli S , Jeffery IB , Santoro A , Neto M , Capri M , Giampieri E , Jennings A , Candela M , Turroni S *et al*. (2020) Mediterranean diet intervention alters the gut microbiome in older people reducing frailty and improving health status: the NU‐AGE 1‐year dietary intervention across five European countries. Gut 69, 1218–1228. 32066625 10.1136/gutjnl-2019-319654 PMC7306987

[259] Mitsou EK , Kakali A , Antonopoulou S , Mountzouris KC , Yannakoulia M , Panagiotakos DB & Kyriacou A (2017) Adherence to the Mediterranean diet is associated with the gut microbiota pattern and gastrointestinal characteristics in an adult population. Br J Nutr 117, 1645–1655. 28789729 10.1017/S0007114517001593

[260] Zhao Y , Jia M , Chen W & Liu Z (2022) The neuroprotective effects of intermittent fasting on brain aging and neurodegenerative diseases via regulating mitochondrial function. Free Radic Biol Med 182, 206–218. 35218914 10.1016/j.freeradbiomed.2022.02.021

[261] Ehrnhoefer DE , Martin DDO , Schmidt ME , Qiu X , Ladha S , Caron NS , Skotte NH , Nguyen YTN , Vaid K , Southwell AL *et al*. (2018) Preventing mutant huntingtin proteolysis and intermittent fasting promote autophagy in models of Huntington disease. Acta Neuropathol Commun 6, 16. 29510748 10.1186/s40478-018-0518-0 PMC5839066

[262] Faris MA , Kacimi S , Al‐Kurd RA , Fararjeh MA , Bustanji YK , Mohammad MK & Salem ML (2012) Intermittent fasting during Ramadan attenuates proinflammatory cytokines and immune cells in healthy subjects. Nutr Res 32, 947–955. 23244540 10.1016/j.nutres.2012.06.021

[263] Wang X , Yang Q , Liao Q , Li M , Zhang P , Santos HO , Kord‐Varkaneh H & Abshirini M (2020) Effects of intermittent fasting diets on plasma concentrations of inflammatory biomarkers: a systematic review and meta‐analysis of randomized controlled trials. Nutrition 79–80, 110974. 10.1016/j.nut.2020.110974 32947129

[264] van Dellen A , Blakemore C , Deacon R , York D & Hannan AJ (2000) Delaying the onset of Huntington's in mice. Nature 404, 721–722. 10783874 10.1038/35008142

[265] Pang TYC , Stam NC , Nithianantharajah J , Howard ML & Hannan AJ (2006) Differential effects of voluntary physical exercise on behavioral and brain‐derived neurotrophic factor expression deficits in Huntington's disease transgenic mice. Neuroscience 141, 569–584. 16716524 10.1016/j.neuroscience.2006.04.013

[266] Novati A , Nguyen HP & Schulze‐Hentrich J (2022) Environmental stimulation in Huntington disease patients and animal models. Neurobiol Dis 171, 105725. 35427742 10.1016/j.nbd.2022.105725

[267] Mertsalmi TH , Pekkonen E & Scheperjans F (2019) Antibiotic exposure and risk of Parkinson's disease in Finland: a nationwide case‐control study. Mov Disord 35, 431–442. 31737957 10.1002/mds.27924

[268] Patangia DV , Anthony Ryan C , Dempsey E , Paul Ross R & Stanton C (2022) Impact of antibiotics on the human microbiome and consequences for host health. Microbiology 11, e1260. 10.1002/mbo3.1260 PMC8756738 35212478

[269] Kim M , Park SJ , Choi S , Chang J , Kim SM , Jeong S , Park YJ , Lee G , Son JS , Ahn JC *et al*. (2022) Association between antibiotics and dementia risk: a retrospective cohort study. Front Pharmacol 13, 888333. 36225572 10.3389/fphar.2022.888333 PMC9548656

[270] Sun J , Zhan Y , Mariosa D , Larsson H , Almqvist C , Ingre C , Zagai U , Pawitan Y & Fang F (2019) Antibiotics use and risk of amyotrophic lateral sclerosis in Sweden. Eur J Neurol 26, 1355–1361. 31087715 10.1111/ene.13986

[271] Chongtham A , Yoo JH , Chin TM , Akingbesote ND , Huda A , Marsh JL & Khoshnan A (2022) Corrigendum: gut bacteria regulate the pathogenesis of Huntington's disease in Drosophila model. Front Neurosci 16, 991513. 36312028 10.3389/fnins.2022.991513 PMC9611772

[272] Sarkar S , Krishna G , Imarisio S , Saiki S , O'Kane CJ & Rubinsztein DC (2007) A rational mechanism for combination treatment of Huntington's disease using lithium and rapamycin. Hum Mol Genet 17, 170–178. 17921520 10.1093/hmg/ddm294

[273] Romero‐Miguel D , Lamanna‐Rama N , Casquero‐Veiga M , Gomez‐Rangel V , Desco M & Soto‐Montenegro ML (2020) Minocycline in neurodegenerative and psychiatric diseases: an update. Eur J Neurol 28, 1056–1081. 33180965 10.1111/ene.14642

[274] Chen M , Ona VO , Li M , Ferrante RJ , Fink KB , Zhu S , Bian J , Guo L , Farrell LA , Hersch SM *et al*. (2000) Minocycline inhibits caspase‐1 and caspase‐3 expression and delays mortality in a transgenic mouse model of Huntington disease. Nat Med 6, 797–801. 10888929 10.1038/77528

[275] Wang X , Zhu S , Drozda M , Zhang W , Stavrovskaya IG , Cattaneo E , Ferrante RJ , Kristal BS & Friedlander RM (2003) Minocycline inhibits caspase‐independent and ‐dependent mitochondrial cell death pathways in models of Huntington's disease. Proc Natl Acad Sci USA 100, 10483–10487. 12930891 10.1073/pnas.1832501100 PMC193587

[276] Stack EC , Smith KM , Ryu H , Cormier K , Chen M , Hagerty SW , Del Signore SJ , Cudkowicz ME , Friedlander RM & Ferrante RJ (2006) Combination therapy using minocycline and coenzyme Q10 in R6/2 transgenic Huntington's disease mice. Biochim Biophys Acta 1762, 373–380. 16364609 10.1016/j.bbadis.2005.11.002

[277] Jones DE Jr , Cui DM & Miller DM (1995) Expression of beta‐galactosidase under the control of the human c‐myc promoter in transgenic mice is inhibited by mithramycin. Oncogene 10, 2323–2330. 7784080

[278] Ferrante RJ , Ryu H , Kubilus JK , D'Mello S , Sugars KL , Lee J , Lu P , Smith K , Browne S , Beal MF *et al*. (2004) Chemotherapy for the brain: the antitumor antibiotic mithramycin prolongs survival in a mouse model of Huntington's disease. J Neurosci 24, 10335–10342. 15548647 10.1523/JNEUROSCI.2599-04.2004 PMC2577231

[279] Koutzoumis DN , Vergara M , Pino J , Buddendorff J , Khoshbouei H , Mandel RJ & Torres GE (2019) Alterations of the gut microbiota with antibiotics protects dopamine neuron loss and improve motor deficits in a pharmacological rodent model of Parkinson's disease. Exp Neurol 325, 113159. 31843492 10.1016/j.expneurol.2019.113159

[280] Lu G , Wen Q , Cui B , Li Q & Zhang F (2022) Washed microbiota transplantation stopped the deterioration of amyotrophic lateral sclerosis: the first case report and narrative review. J Biomed Res 37, 69–76. 35821195 10.7555/JBR.36.20220088 PMC9898040

[281] Minter MR , Zhang C , Leone V , Ringus DL , Zhang X , Oyler‐Castrillo P , Musch MW , Liao F , Ward JF , Holtzman DM *et al*. (2016) Antibiotic‐induced perturbations in gut microbial diversity influences neuro‐inflammation and amyloidosis in a murine model of Alzheimer's disease. Sci Rep 6, 30028. 27443609 10.1038/srep30028 PMC4956742

[282] Yan H , Walker FC , Ali A , Han H , Tan L , Veillon L , Lorenzi PL , Baldridge MT & King KY (2022) The bacterial microbiota regulates normal hematopoiesis via metabolite‐induced type 1 interferon signaling. Blood Adv 6, 1754–1765. 35143611 10.1182/bloodadvances.2021006816 PMC8941453

[283] Josefsdottir KS , Baldridge MT , Kadmon CS & King KY (2017) Antibiotics impair murine hematopoiesis by depleting the intestinal microbiota. Blood 129, 729–739. 27879260 10.1182/blood-2016-03-708594 PMC5301822

[284] Lewis SM , Williams A & Eisenbarth SC (2019) Structure and function of the immune system in the spleen. Sci Immunol 4, eaau6085. 30824527 10.1126/sciimmunol.aau6085 PMC6495537

[285] Zhu H , Liu Y , Li S , Jin Y , Zhao L , Zhao F , Feng J , Yan W & Wei Y (2018) Altered gut microbiota after traumatic splenectomy is associated with endotoxemia. Emerg Microbes Infect 7, 197. 30498207 10.1038/s41426-018-0202-2 PMC6265257

[286] Wei Y , Chang L , Ishima T , Wan X , Ma L , Wuyun G , Pu Y & Hashimoto K (2021) Abnormalities of the composition of the gut microbiota and short‐chain fatty acids in mice after splenectomy. Brain Behav Immun Health 11, 100198. 34589731 10.1016/j.bbih.2021.100198 PMC8474575

[287] Heemskerk AW & Roos RA (2012) Aspiration pneumonia and death in Huntington's disease. PLoS Curr 4, RRN1293. 22307361 10.1371/currents.RRN1293 PMC3269785

[288] Carreno D , Wanford JJ , Jasiunaite Z , Hames RG , Chung WY , Dennison AR , Straatman K , Martinez‐Pomares L , Pareek M , Orihuela CJ *et al*. (2021) Splenic macrophages as the source of bacteraemia during pneumococcal pneumonia. EBioMedicine 72, 103601. 34619637 10.1016/j.ebiom.2021.103601 PMC8498229

[289] Mok SW , Wong VK , Lo HH , de Seabra Rodrigues Dias IR , Leung EL , Law BY & Liu L (2020) Natural products‐based polypharmacological modulation of the peripheral immune system for the treatment of neuropsychiatric disorders. Pharmacol Ther 208, 107480. 31972182 10.1016/j.pharmthera.2020.107480

[290] You Z , Luo C , Zhang W , Chen Y , He J , Zhao Q , Zuo R & Wu Y (2011) Pro‐ and anti‐inflammatory cytokines expression in rat's brain and spleen exposed to chronic mild stress: involvement in depression. Behav Brain Res 225, 135–141. 21767575 10.1016/j.bbr.2011.07.006

[291] Wan X , Eguchi A , Sakamoto A , Fujita Y , Yang Y , Qu Y , Hatano M , Mori C & Hashimoto K (2022) Impact of broad‐spectrum antibiotics on the gut‐microbiota‐spleen‐brain axis. Brain Behav Immun Health 27, 100573. 36583066 10.1016/j.bbih.2022.100573 PMC9793168

[292] Kolypetri P , Liu S , Cox LM , Fujiwara M , Raheja R , Ghitza D , Song A , Daatselaar D , Willocq V & Weiner HL (2021) Regulation of splenic monocyte homeostasis and function by gut microbial products. iScience 24, 102356. 33898947 10.1016/j.isci.2021.102356 PMC8059056

[293] Blomster LV , Brennan FH , Lao HW , Harle DW , Harvey AR & Ruitenberg MJ (2013) Mobilisation of the splenic monocyte reservoir and peripheral CX_3_CR1 deficiency adversely affects recovery from spinal cord injury. Exp Neurol 247, 226–240. 23664962 10.1016/j.expneurol.2013.05.002

[294] Butovsky O , Siddiqui S , Gabriely G , Lanser AJ , Dake B , Murugaiyan G , Doykan CE , Wu PM , Gali RR , Iyer LK *et al*. (2012) Modulating inflammatory monocytes with a unique microRNA gene signature ameliorates murine ALS. J Clin Invest 122, 3063–3087. 22863620 10.1172/JCI62636 PMC3428086

[295] Yan P , Kim KW , Xiao Q , Ma X , Czerniewski LR , Liu H , Rawnsley DR , Yan Y , Randolph GJ , Epelman S *et al*. (2022) Peripheral monocyte‐derived cells counter amyloid plaque pathogenesis in a mouse model of Alzheimer's disease. J Clin Invest 132, e152565. 35511433 10.1172/JCI152565 PMC9151689

[296] Grozdanov V , Bliederhaeuser C , Ruf WP , Roth V , Fundel‐Clemens K , Zondler L , Brenner D , Martin‐Villalba A , Hengerer B , Kassubek J *et al*. (2014) Inflammatory dysregulation of blood monocytes in Parkinson's disease patients. Acta Neuropathol 128, 651–663. 25284487 10.1007/s00401-014-1345-4 PMC4201759

[297] Zheng X , Ma S , Kang A , Wu M , Wang L , Wang Q , Wang G & Hao H (2016) Chemical dampening of Ly6Chi monocytes in the periphery produces anti‐depressant effects in mice. Sci Rep 6, 19406. 26783261 10.1038/srep19406 PMC4725984

[298] van de Wouw M , Boehme M , Dinan TG & Cryan JF (2019) Monocyte mobilisation, microbiota & mental illness. Brain Behav Immun 81, 74–91. 31330299 10.1016/j.bbi.2019.07.019

[299] Jones GR , Bain CC , Fenton TM , Kelly A , Brown SL , Ivens AC , Travis MA , Cook PC & MacDonald AS (2018) Dynamics of colon monocyte and macrophage activation during colitis. Front Immunol 9, 2764. 30542349 10.3389/fimmu.2018.02764 PMC6277765

[300] Swirski FK , Nahrendorf M , Etzrodt M , Wildgruber M , Cortez‐Retamozo V , Panizzi P , Figueiredo JL , Kohler RH , Chudnovskiy A , Waterman P *et al*. (2009) Identification of splenic reservoir monocytes and their deployment to inflammatory sites. Science 325, 612–616. 19644120 10.1126/science.1175202 PMC2803111

[301] Raghavan S , Singh NK , Gali S , Mani AM & Rao GN (2018) Protein kinase Cθ via activating transcription factor 2‐mediated CD36 expression and foam cell formation of Ly6C(hi) cells contributes to atherosclerosis. Circulation 138, 2395–2412. 29991487 10.1161/CIRCULATIONAHA.118.034083 PMC6309268

[302] Fischer M , Sipe B , Cheng YW , Phelps E , Rogers N , Sagi S , Bohm M , Xu H & Kassam Z (2016) Fecal microbiota transplant in severe and severe‐complicated *Clostridium difficile*: a promising treatment approach. Gut Microbes 8, 289–302. 28001467 10.1080/19490976.2016.1273998 PMC5479393

[303] Spartz EJ , Estafanos M , Mallick R , Gaertner W , Vakayil V , Jahansouz C , Aggarwal R , Ikramuddin S , Khoruts A & Harmon JV (2023) Fecal microbiota transplantation for fulminant *Clostridioides difficile* infection: a combined medical and surgical case series. Cureus 15, e34998. 36938160 10.7759/cureus.34998 PMC10020130

[304] Feuerstadt P , Louie TJ , Lashner B , Wang EEL , Diao L , Bryant JA , Sims M , Kraft CS , Cohen SH , Berenson CS *et al*. (2022) SER‐109, an oral microbiome therapy for recurrent *Clostridioides difficile* infection. N Engl J Med 386, 220–229. 35045228 10.1056/NEJMoa2106516

[305] Khanna S , Assi M , Lee C , Yoho D , Louie T , Knapple W , Aguilar H , Garcia‐Diaz J , Wang GP , Berry SM *et al*. (2022) Efficacy and safety of RBX2660 in PUNCH CD3, a phase III, randomized, double‐blind, placebo‐controlled trial with a Bayesian primary analysis for the prevention of recurrent *Clostridioides difficile* infection. Drugs 82, 1527–1538. 36287379 10.1007/s40265-022-01797-x PMC9607700

[306] Chinna Meyyappan A , Forth E , Wallace CJK & Milev R (2020) Effect of fecal microbiota transplant on symptoms of psychiatric disorders: a systematic review. BMC Psychiatry 20, 299. 32539741 10.1186/s12888-020-02654-5 PMC7294648

[307] Takacova M , Bomba A , Tothova C , Michalova A & Turna H (2022) Any future for faecal microbiota transplantation as a novel strategy for gut microbiota modulation in human and veterinary medicine? Life (Basel) 12, 723. 35629390 10.3390/life12050723 PMC9146664

[308] Xu HM , Huang HL , Zhou YL , Zhao HL , Xu J , Shou DW , Liu YD , Zhou YJ & Nie YQ (2021) Fecal microbiota transplantation: a new therapeutic attempt from the gut to the brain. Gastroenterol Res Pract 2021, 6699268. 33510784 10.1155/2021/6699268 PMC7826222

[309] McOmish CE & Hannan AJ (2007) Enviromimetics: exploring gene environment interactions to identify therapeutic targets for brain disorders. Expert Opin Ther Targets 11, 899–913. 17614759 10.1517/14728222.11.7.899

[310] Gubert C & Hannan AJ (2021) Exercise mimetics: harnessing the therapeutic effects of physical activity. Nat Rev Drug Discov 20, 862–879. 34103713 10.1038/s41573-021-00217-1

[311] Ellrichmann G , Blusch A , Fatoba O , Brunner J , Reick C , Hayardeny L , Hayden M , Sehr D , Winklhofer KF , Saft C *et al*. (2017) Laquinimod treatment in the R6/2 mouse model. Sci Rep 7, 4947. 28694434 10.1038/s41598-017-04990-1 PMC5504033

[312] Carroll JB , Warby SC , Southwell AL , Doty CN , Greenlee S , Skotte N , Hung G , Bennett CF , Freier SM & Hayden MR (2011) Potent and selective antisense oligonucleotides targeting single‐nucleotide polymorphisms in the Huntington disease gene/allele‐specific silencing of mutant huntingtin. Mol Ther 19, 2178–2185. 21971427 10.1038/mt.2011.201 PMC3242664

[313] Gray M , Shirasaki DI , Cepeda C , Andre VM , Wilburn B , Lu XH , Tao J , Yamazaki I , Li SH , Sun YE *et al*. (2008) Full‐length human mutant huntingtin with a stable polyglutamine repeat can elicit progressive and selective neuropathogenesis in BACHD mice. J Neurosci 28, 6182–6195. 18550760 10.1523/JNEUROSCI.0857-08.2008 PMC2630800

[314] Mears ER , Handley RR , Grant MJ , Reid SJ , Day BT , Rudiger SR , McLaughlan CJ , Verma PJ , Bawden SC , Patassini S *et al*. (2021) A multi‐omic huntington's disease transgenic sheep‐model database for investigating disease pathogenesis. J Huntingtons Dis 10, 423–434. 34420978 10.3233/JHD-210482 PMC8673501

[315] Ferrante RJ (2009) Mouse models of Huntington's disease and methodological considerations for therapeutic trials. Biochim Biophys Acta 1792, 506–520. 19362590 10.1016/j.bbadis.2009.04.001 PMC2693467

